# β‐Asarone Attenuates Neuroinflammation of Alzheimer's Disease by Activating Autophagy and Suppressing NLRP3 Inflammasome Assembly

**DOI:** 10.1002/cns.70771

**Published:** 2026-02-04

**Authors:** Zhiwei Xu, Wanying Xu, Jinxin He, Jiahui Qian, Hui Wang, Changyu Li, Xiaojie Zhou

**Affiliations:** ^1^ Jinhua Academy Zhejiang Chinese Medical University Jinhua China; ^2^ Second Clinical Medical School Zhejiang Chinese Medical University Hangzhou China; ^3^ School of Pharmaceutical Sciences Zhejiang Chinese Medical University Hangzhou China; ^4^ Academy of Chinese Medical Sciences Zhejiang Chinese Medical University Hangzhou China

**Keywords:** β‐Asarone, Alzheimer's disease, autophagy, neuroinflammation, NLRP3 inflammasome

## Abstract

**Aim:**

Alzheimer's Disease (AD) is a neurodegenerative condition with poorly understood mechanisms and few effective treatments. β‐asarone has shown potential in AD management, though its molecular actions require further clarification. This study investigates the mechanisms through which β‐asarone exerts its effects using both animal and cellular models.

**Methods:**

In vivo, the 3×Tg‐AD mice were administered β‐asarone for 8 weeks. Learning and memory abilities were assessed via the Morris water maze and step‐down tests. Histomorphological examination, immunofluorescence, immunohistochemistry, ELISA, transmission electron microscopy, and Western blotting were employed to detect pathological changes, neuroinflammation, and protein expression of relevant signaling pathway molecules. In vitro, Aβ was used to culture BV‐2 cells to mimic the brain microenvironment in Alzheimer's disease; changes in neuroinflammation, autophagy, and NLRP3 inflammasome‐related proteins were observed after treatment with β‐asarone.

**Results:**

The administration of β‐asarone resulted in enhanced cognitive performance in 3×Tg‐AD mice, alongside a reduction in microglial apoptosis induced by Aβ. Additionally, β‐asarone diminished the accumulation of Aβ and phosphorylated Tau, ultimately supporting neuronal survival. In both the hippocampal tissue and BV‐2 cell models, treatment with β‐asarone led to a downregulation of neuroinflammatory markers and modulation of autophagy‐related proteins (Beclin‐1, P62, ATG5, LC3‐II/I), while concurrently suppressing components of the NLRP3 inflammasome (NLRP3, ASC, Caspase‐1, cleaved Caspase‐1). Notably, the autophagy inhibitor 3‐MA counteracted the inhibitory effects of β‐asarone on NLRP3 activation.

**Conclusion:**

β‐Asarone attenuates AD‐related neuroinflammation by activating autophagy to inhibit NLRP3 inflammasome assembly.

## Graphical abstract

β‐amyloid activates microglia via the TLR/NF‐κB pathway and primes the NLRP3 inflammasome, driving neuroinflammation. β‐asarone counteracts this by activating autophagy, which degrades NLRP3 components and inhibits inflammasome assembly. This reduces IL‐1β maturation, suppresses cytokine release, and alleviates neuronal damage and cognitive deficits in Alzheimer's models.

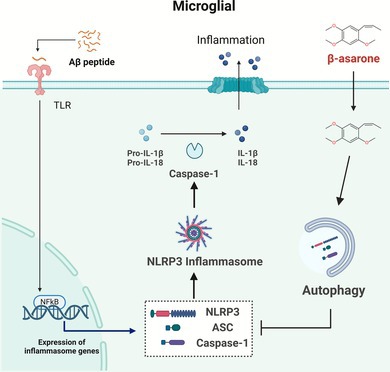

## 1 Introduction

Alzheimer's disease (AD) is a prevalent and progressive neurodegenerative condition. The pathological features of AD are typified by the accumulation of extracellular amyloid‐beta (Aβ) plaques, intracellular neurofibrillary tangles formed by hyperphosphorylated Tau protein, and a chronic neuroinflammatory state [1]. Current estimates indicate that approximately 55 million individuals are afflicted with AD globally, and the annual healthcare expenditures can reach trillions of dollars. With the aging population, there is a significant rise in both the prevalence of AD patients and associated medical expenses [2]. Currently, the approved treatment involves cholinesterase inhibitors, NMDA‐receptor antagonists, and their combination. However, these drugs used in clinic are difficult to fundamentally treat AD [3, 4]. Hence, it is imperative to deepen investigation into the mechanisms and therapeutics of AD. Current research on AD pathogenesis primarily centers on the hypotheses of Aβ deposition, Tau hyperphosphorylation, neuroinflammatory activation, and autophagic dysfunction [5]. Among these, neuroinflammation and autophagy have received increasing attention from researchers [6]. The NLRP3‐mediated inflammatory response in microglial cells is pivotal in the development of neuroinflammation [7]. Autophagy serves to mitigate excessive inflammatory responses by inhibiting the formation of the NLRP3 inflammasome, which subsequently leads to a reduction in Aβ accumulation and tau hyperphosphorylation [8, 9]. Therefore, targeting the inhibition of NLRP3 inflammasome formation via the activation of microglial autophagy may represent a significant strategy in the management of AD.

β‐Asarone is a key active component of *Acorus tatarinowii* Schott, a plant with a well‐documented history in traditional Chinese medicine (TCM) for treating neurological disorders. Numerous studies have shown that TCM can effectively alleviates clinical symptoms in early Alzheimer's disease patients [10, 11, 12]. Therefore, it is a very promising approach to explore efficient and low‐toxicity compounds from traditional Chinese medicine for the treatment of AD. *Acorus tatarrinowii* Schott, the dried rhizome of *Acorus tatarrinowii* Schott, is a representative Chinese medicine for the prevention and treatment of AD. It is widely used in clinical treatment of senile dementia, fornication, epilepsy, and other diseases [13]. Our previous experiments confirmed that the volatile oil of Acorus acorus is the main pharmacodynamic site in the treatment of AD, among which β‐asarone is the main chemical component of the volatile oil of Acorus acorus [14, 15]. Research indicates that β‐asarone affects multiple Alzheimer's disease‐related targets by enhancing neuronal protection, promoting BDNF synthesis and release, inhibiting Aβ aggregation, reducing the formation of Aβ plaques, and encouraging neuronal regeneration [16, 17]. However, it has not been reported whether it can reduce neuroinflammation by activating autophagy through inhibiting the formation of NLRP3 inflammasome.

In this study, our results demonstrated that β‐asarone effectively diminishes neuroinflammation in AD mouse models. Furthermore, β‐asarone was found to inhibit NLRP3 inflammasome formation by activating microglial autophagy, leading to a reduction in neuroinflammation.

## 2 Materials and Methods

### 2.1 Drug and Reagents

The β‐asarone was purchased from Shanghai Yuanye Biotechnology Co. LTD. (Specification: 500 mg, batch number: J22HB174800, ≥ 98%). Donepezil (Lot number: 1912024) was sourced from Eisai Pharmaceutical Co. Ltd. (Suzhou, China). Saline (Lot number: 2021031001) was obtained from Anhui Jiutian Yingnuo Animal Pharmaceutical Co. LTD (Tongling, China). Lipopolysaccharide (LPS) was obtained from Shanghai Yuanye Biotechnology Co. LTD (Lot number: S11060).

### 2.2 Animal Grouping and Treatment

A total of thirty 12‐month‐old male 3×Tg‐AD mice were utilized in this experiment. Prior to group assignment, cognitive impairments were validated in all transgenic subjects using the Morris water maze (MWM) assessment. The mice were then randomly allocated into three experimental groups (*n* = 10 per group): the 3×Tg‐AD group, the donepezil group (2 mg/kg) [18], and the β‐asarone group (30 mg/kg). An additional cohort of ten wild‐type B6 mice served as the normal control (WT) group. Both the WT and 3×Tg‐AD control groups were administered saline, while the drug treatments were conducted daily over a period of 8 weeks.

### 2.3 Cell Culture and Treatment

In this investigation, BV‐2 cells were employed. To establish an in vitro model of amyloid‐β‐induced neurotoxicity, the BV‐2 cells were treated with 25 μM Aβ_25–35_ peptide (MedChemExpress, Shanghai, China) for 24 h [19, 20, 21]. Cells were exposed to a range of β‐asarone concentrations (5, 10, 20, 40, 80, 160, 320 μg/mL) to identify the most effective concentration. Afterward, cells were split into the following categories: control (cells maintained in DMEM only), Aβ group (cells cultured with Aβ_25–35_), β‐asarone group (different doses of β‐asarone were given for 12 h after Aβ_25‐35_ intervention), 3‐MA (3‐Methyladenine) group (β‐asarone and 10 μM 3‐MA were treated simultaneously for 12 h after Aβ_25–35_ intervention) [22, 23].

### 2.4 Behavioral Test

#### 2.4.1 Morris Water Maze Test (MWM)

Prior to the experimental procedure, mice were acclimatized to the Morris Water Maze (MWM) for 1 day. During the subsequent 5 days, their navigational abilities towards a submerged platform were evaluated. In this phase, the mice were placed into the water to locate the hidden platform, with each mouse undergoing a 60‐s training session in each quadrant. After this training phase, the platform was removed, and each mouse was positioned in the maze from the quadrant opposite to the initial platform location. The parameters recorded during the 60‐s probe trial included swimming trajectory, latency to the previously occupied platform location, number of platform crossings, as well as the total distance and time spent in the target quadrant.

#### 2.4.2 Step‐Down Test

The mice were acclimated for 5 min on the mouse platform recorder. Following the 4‐h interval, mice were re‐exposed to the platform to undergo a conditioned training trial, during which foot shocks were delivered via the activated electrical stimulator. Mice received a shock to their limbs upon jumping off the platform, prompting them to return to avoid further shocks. The mice were reintroduced to the platform 24 h post‐training and observed for a duration of 5 min. Parameters recorded included the latency to the first jump and the number of jumps (errors).

### 2.5 Cell Counting Kit‐8 (CCK‐8) Assay

Cells in the logarithmic growth phase were harvested and seeded in 96‐well plates at a density of 1.0 × 10^5^ cells per well in 100 μL. Upon reaching 50%–70% confluence, cells were starved in serum‐free medium for 2 h before being treated with varying concentrations of drugs. The experimental procedures were conducted in accordance with the manufacturer's instructions provided in the CCK8 manual.

### 2.6 Flow Cytometry

Cells (0.5 ~ 1 × 10^6^) were collected and washed twice with PBS, adding 100 μL Binding Buffer and FITC‐labeled Annexin‐V (20 μg/mL) 10 μL at room temperature for 30 min, and then adding PI (50 μg/mL) 5 μL. After 5 min of light shielding reaction, 400 μL Binding Buffer was added, and flow cytometry was performed for quantitative detection by FACScan immediately. Meanwhile, one tube without AnnexinV‐FITC and PI was used as negative control.

### 2.7 Histological Analysis of Brain Sections

Brain tissues were fixed in 4% paraformaldehyde for 48 h and subsequently processed through graded ethanol and xylene series prior to paraffin embedding. Sections of approximately 4 μm thickness were prepared using a microtome and then dewaxed. Hematoxylin and eosin (HE) staining and Nissl staining were conducted on the paraffin‐embedded sections for histological evaluation under a light microscope. The degree of neural injury was assessed by calculating the denatured cell index (DCI) from HE‐stained images, defined as (degenerated cells/total cells) × 100. Additionally, the density of viable neurons containing Nissl bodies was quantified within a standardized field of view.

### 2.8 Immunofluorescence

The paraffin sections underwent dewaxing using varying concentrations of ethanol and xylene, followed by subjecting them to antigen repair in preheated EDTA solution. For cellular immunofluorescence, slides were placed in 24‐well plates and cells were seeded. Different groups were treated with different methods. Each well was fixed with 4% paraformaldehyde solution and permeabilized with Triton X‐100. 3% BSA was used to block the paraffin sections and cells. Then, the blocked paraffin sections and cell slides were added with a specific primary antibody. The primary antibodies included NeuN (neuronal nuclei) (1:200 Abcam, UK), Aβ (1:200, CST, USA), and Iba‐1 (ionized calcium binding adapter molecule 1) antibody (1:200, CST, USA). Subsequently, the paraffin sections and cell slides were incubated with the corresponding secondary antibody. The images were captured by Zeiss upright fluorescence microscope.

### 2.9 Immunohistochemistry

Paraffin sections were dewaxed using varying concentrations of ethanol and xylene, followed by incubating with 3% H_2_O_2_ to eliminate endogenous peroxidase activity. Then the paraffin sections perform antigen repair by preheated EDTA solution. Subsequently, they were blocked with 3% BSA and incubated with Iba‐1 antibody (1:200, CST, USA). DAB kit (Beyotime, Beijing, China) was used to visualize the immunoreactivity. Sections were observed by the light microscope.

### 2.10 Fluoro‐Jade B Staining

Brain sections were stained with Fluoro‐Jade B (FJB) (Merck, Germany) to label degenerating neurons. Briefly, after deparaffinization and rehydration, slides were oxidized with 0.06% KMnO_4_, rinsed, and then incubated in 0.001% FJB (in 0.1% acetic acid) for 30 min. After washing, slides were dried, cleared in xylene, and coverslipped with DPX. Images were captured using a fluorescence microscope with a FITC filter set.

### 2.11 mRFP‐GFP‐LC3 Adenovirus Analysis

To assess autophagic flux, cells were infected with a recombinant adenovirus encoding tandem fluorescent‐tagged LC3 (mRFP‐GFP‐LC3). Briefly, cells were plated and infected at a multiplicity of infection (MOI) of 10 for optimal expression. After 24 h, the medium was replaced, and cells were allowed to express the construct for an additional 24 h. Live‐cell imaging was performed using a laser scanning confocal microscope. GFP (pH‐sensitive) and mRFP (pH‐stable) signals were captured simultaneously. Autophagic compartments were quantified: yellow puncta (mRFP and GFP colocalization) represent autophagosomes, while red‐only puncta (mRFP signal without GFP) indicate autolysosomes where GFP fluorescence is quenched in the acidic lumen.

### 2.12 Transmission Electron Microscopy

For ultrastructural analysis of autophagic vacuoles, cells were primarily fixed with 2.5% glutaraldehyde in 0.1 M phosphate buffer, followed by post‐fixation in 1% osmium tetroxide. After dehydration through a graded ethanol series, samples were embedded in epoxy resin. Ultrathin sections were cut, stained with uranyl acetate and lead citrate, and examined under a transmission electron microscope (TEM, Hitachi H‐7650). Autophagic structures, including autophagosomes (double‐membrane vesicles containing cytoplasmic material) and autolysosomes (single‐membrane vesicles with degraded contents), were identified and quantified per cellular profile.

### 2.13 Western Blot Analysis

The hippocampal tissue frozen at −80°C was used for protein extraction. All protein analyses were performed on RIPA‐soluble fractions, and the protein concentration of each sample was detected by BCA protein quantitative kit (Beyotime, Beijing, China). SDS‐PAGE gels of varying concentrations were chosen based on the molecular weights of the target proteins. A 30 μg protein sample was subjected to SDS‐PAGE at 120 V for 60 min, followed by transfer to a PVDF membrane at 350 mA for 90 min. Following protein transfer, membranes were blocked with 5% BSA at room temperature for 1 h, then incubated with a specific primary antibody. The primary antibodies used were NeuN, PSD95 (postsynaptic density protein 95), Beclin‐1 (myosin‐Like Bcl‐2 interacting protein), P62 (sequestosome‐1, SQSTM1), and ATG5 (autophagy‐related proteins 5) from Abcam (UK); Tau (1:1000, Thermo Scientific, reactive to human tau; epitope: aa 159–163); Phosphorylated tau at Ser396 was detected with phospho‐specific antibody (1:1000, Immunoway PS396‐1). Iba‐1, NLRP3 (nucleotide binding domain and leucine‐rich repeat protein 3), ASC (apoptosis‐associated speck‐like protein containing CARD), Caspase‐1 (Cysteine‐dependent aspartate‐specific protease), and LC3 (microtubule‐associated protein 1 light chain 3) from CST (USA); and GFAP (glial fibrillary acidic protein) from Huaan Biotechnology (China), all at a dilution of 1:1000. Membranes were then incubated with an HRP‐conjugated secondary antibody (1:1000, Abcam, UK) for 2 h at 37°C and visualized using an ECL chemiluminescent kit (Thermo Fisher, Shanghai, China).

### 2.14 Statistical Analysis

Statistical analyses were performed using SPSS 24.0 and GraphPad Prism 8.0. Normality of the data was assessed using the Shapiro–Wilk test (for sample sizes *n* < 50). For data conforming to a normal distribution (*p* > 0.05), one‐way ANOVA followed by Tukey's post hoc test was employed. When the assumptions of parametric tests are not satisfied, the Kruskal–Wallis test will be applied for statistical analysis. Results are expressed as mean ± standard deviation (SD). Statistical significance was defined as *p* < 0.05.

## 3 Results

### 3.1 β‐Asarone Treatment Improved Cognitive Function in 3×Tg‐AD Mice

**FIGURE 1:**
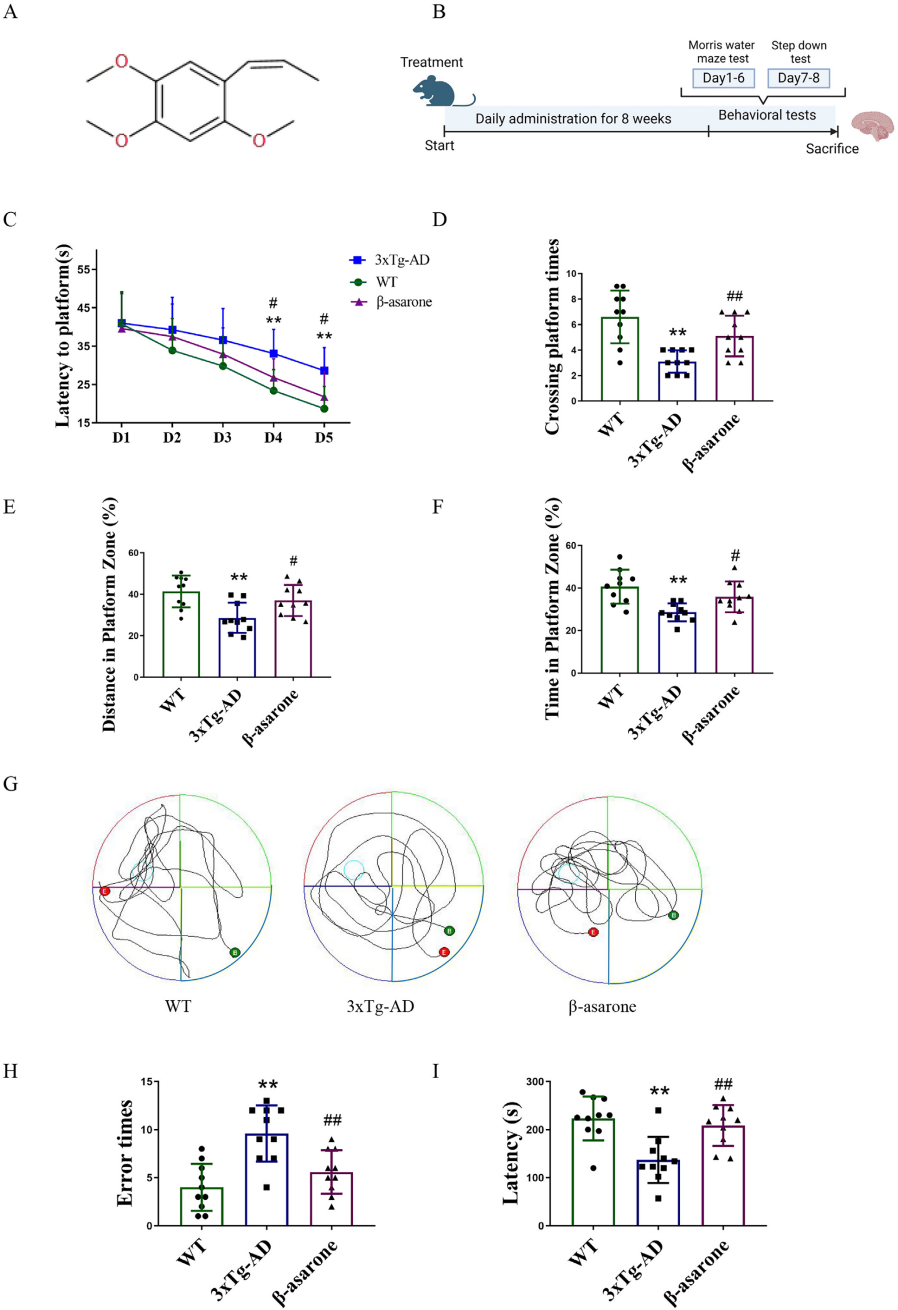
β‐asarone enhanced the learning and cognitive functions in 3×Tg‐AD mice. (A) The chemical structure of β‐asarone. (B) The schedule of animal experiments. (C–F) The latency, crossing platform times, distance in platform zoom, and swimming track map of mice in MWM. (H, I) The error times (incorrect platform descents) and latency (first descent latency) of mice in step‐down test. MWM pool diameter (120 cm), platform size (10 cm), trial duration (60 s). Data are expressed as mean ± SD. Statistics: One‐way ANOVA with Tukey's test. *p < 0.05, **p < 0.01, relative to WT group; ^#^ *p* < 0.05, ^##^ *p* < 0.01, relative to 3×Tg‐AD group. *n* = 10/group.

The chemical structure of β‐asarone is shown in Figure 1A. The experimental procedure and drug administration details are illustrated in Figure 1B. The Morris Water Maze (MWM) and the step‐down test are widely utilized behavioral methods for assessing cognitive function in mice [24]. As illustrated in Figure 1C, β‐asarone administration significantly reduced the escape latency relative to the 3×Tg‐AD group, with a statistically notable difference appearing by the fourth day. Compared to wild‐type (WT) controls, 3×Tg‐AD mice displayed prolonged latency to locate the platform, an impairment that was markedly alleviated by β‐asarone treatment. Furthermore, parameters including number of platform crossings, path length within the target zone, and time spent in the target quadrant were all significantly decreased in 3×Tg‐AD mice. β‐asarone treatment notably reversed these deficits (Figure 1D–F). Representative swimming paths further confirmed that mice receiving β‐asarone located the platform more rapidly and directly than those in the 3×Tg‐AD group (Figure 1G). Consistent with these findings, the step‐down test revealed a significant reduction in error counts and an increase in step‐down latency following β‐asarone intervention (Figure 1H,I). Collectively, these results indicate that β‐asarone treatment contributes to the enhancement of cognitive function in 3×Tg‐AD mice.

### 3.2 β‐Asarone Treatment Ameliorated Pathological Injury and Neuron Loss in the Hippocampus of 3×Tg‐AD Mice

**FIGURE 2:**
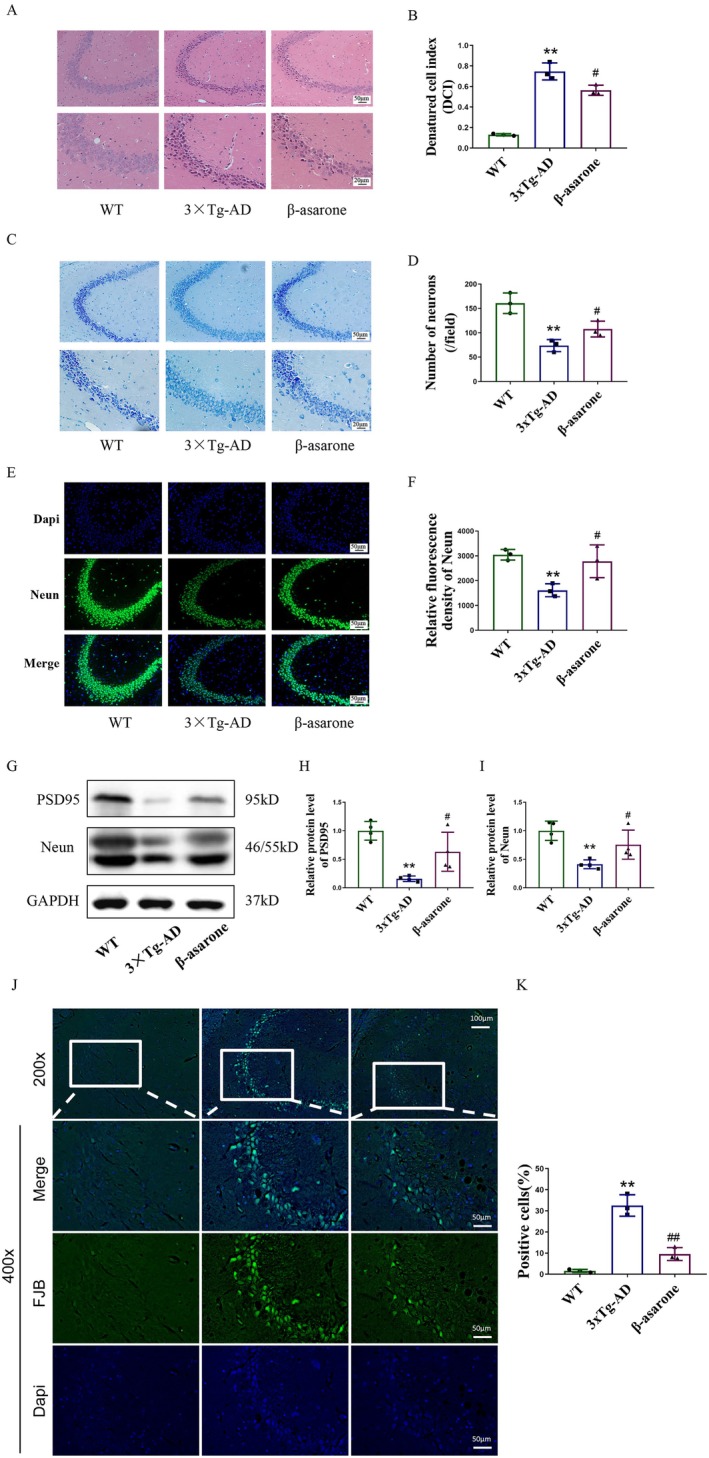
β‐asarone mitigated hippocampal damage and neuronal loss in hippocampus of 3×Tg‐AD mice. (A, C) Representative *HE* staining and Nissl staining of CA3 area in the hippocampus (200×, 400×). (B, D) Quantification of denatured cells and Nissl‐positive neurons. Denatured Cell Index (DCI) = (degenerated neurons/total neurons) × 100%. Counted from 5 random fields/section. (E) Representative immunofluorescent images of Neun (green)/DAPI (blue) colocalization (200×). (F) Quantification of relative fluorescence density of Neun. (G) Representative immunoblots of PSD95 and Neun. (H, I) Quantification of relative protein level of PSD95 and Neun. (J) Representative images of Fluoro‐Jade B staining. (K) Quantification of positive cells. Statistics: One‐way ANOVA with Tukey's test. Data are expressed as mean ± SD. *p < 0.05, **p < 0.01, relative to WT group; ^#^ *p* < 0.05, ^##^ *p* < 0.01, relative to 3×Tg‐AD group. *n* = 3/group.

The results of HE staining are presented in Figure 2A. In the CA3 area of the hippocampus from WT mice, the tissue displayed considerable thickness and high cellular density, with neurons arranged in a neat and closely packed manner. The morphology of the cells appeared regular, and the structural integrity was maintained, with a clear boundary evident. In contrast, hippocampal neurons in 3×Tg‐AD mice exhibited disordered arrangements, irregular morphologies, increased spacing between neurons, and intensified cytopathological changes.

Immunofluorescence and western blot were used to detect the expression of Neun, the results showed that Neun in the hippocampus of mice in the 3×Tg‐AD group was significantly decreased, and the expression could be improved after the intervention of β‐asarone (Figure 2E–G,I). Meanwhile, PSD95 was detected by western blot, the result shown in Figure 2G,H, the expression in the hippocampus of mice in the 3×Tg‐AD group was decreased markedly and could be increased after the intervention of β‐asarone. As shown in Figure 2J,K, the model group exhibited a marked increase in the density of FJB‐positive cells. In contrast, this increase was significantly attenuated by drug treatment, which resulted in a marked reduction in positive cell density. These results suggested that. β‐asarone treatment significantly ameliorated pathological damage and neuron loss in the hippocampus of 3×Tg‐AD mice.

### 3.3 β‐Asarone Treatment Ameliorated Characteristic Protein Expression of AD in the Hippocampus of 3×Tg‐AD Mice

**FIGURE 3:**
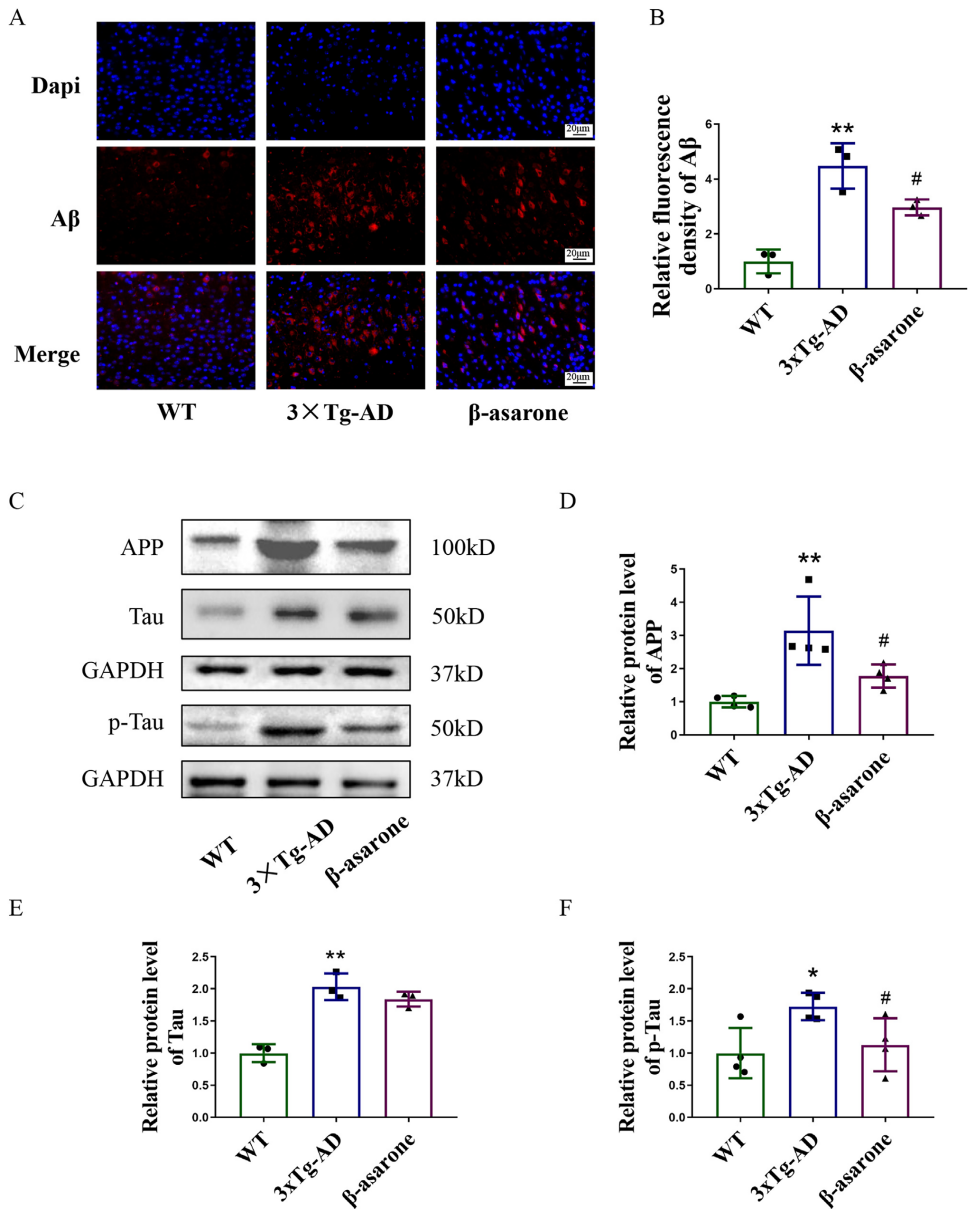
β‐asarone ameliorated characteristic protein expression of AD in hippocampus of 3×Tg‐AD mice. (A) Representative immunofluorescent images of Aβ (red)/DAPI (blue) colocalization in the hippocampus of mice (400×). (B) Quantification of relative fluorescence density of Aβ. (C) Representative immunoblots of APP, Tau and p‐Tau. (D–F) Quantification of relative protein level of APP, Tau and p‐Tau. Data are expressed as mean ± SD. Statistics: One‐way ANOVA with Tukey's test. *p < 0.05, **p < 0.01, relative to WT group; ^#^ *p* < 0.05, ^##^ *p* < 0.01, relative to 3×Tg‐AD group. *n* = 3/group.

The deposition of Aβ and the hyperphosphorylation of Tau protein are hallmark features of AD. The precursor protein for Aβ production, amyloid‐beta precursor protein (APP), was evaluated through immunofluorescence, with results depicted in Figure 3A,B. The expression levels of APP and Tau phosphorylation were analyzed utilizing Western blot techniques, with outcomes presented in Figure 3C–F. Our findings demonstrated that β‐asarone treatment led to a reduction in the expression of Aβ, APP, and Tau phosphorylation, thereby indicating that β‐asarone ameliorates the characteristic protein expression associated with AD in 3×Tg‐AD mice.

### 3.4 β‐Asarone Treatment Alleviated the Neuroinflammation in the Hippocampus of 3×Tg‐AD Mice

**FIGURE 4:**
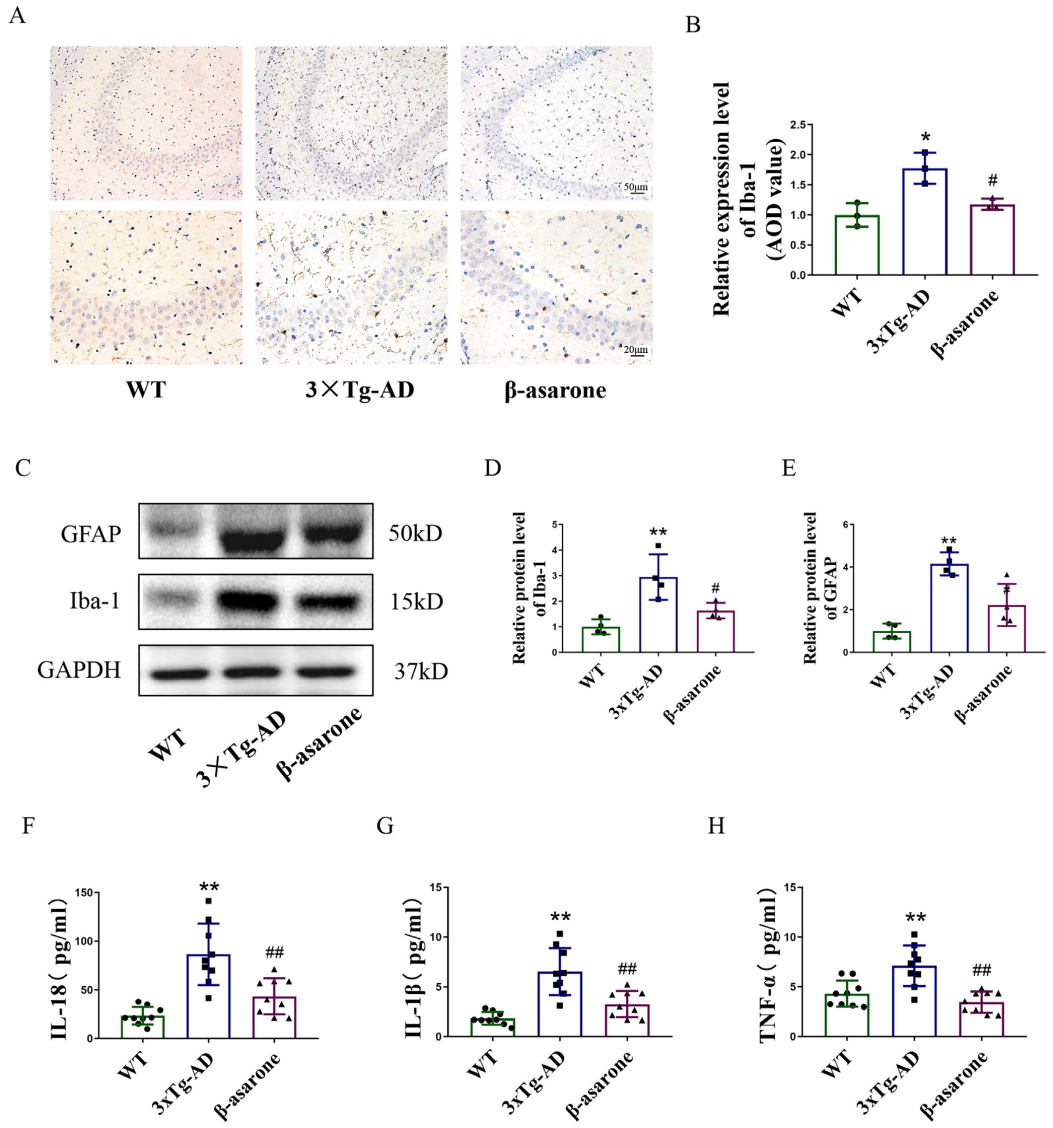
β‐asarone alleviated the neuroinflammation in hippocampus of 3×Tg‐AD mice. (A) Representative immunohistochemical images of Iba‐1. (B) Quantification of Iba‐1 positive cells (*n* = 3). (C) Representative immunoblots of GFAP and Iba‐1. (D, E) Quantification of relative protein level of GFAP and Iba‐1 (*n* = 3). (F–H) ELISA of hippocampal IL‐18, IL‐1β, and TNF‐α levels (*n* = 9). Data are expressed as mean ± SD. Statistics: One‐way ANOVA with Tukey's test. *p < 0.05, **p < 0.01, relative to WT group; ^#^ *p* < 0.05, ^##^ *p* < 0.01, relative to 3×Tg‐AD group. *n* = 3/group.

Neuroinflammation plays a critical role in AD, contributing to neuronal loss, Aβ accumulation, and tau hyperphosphorylation. Immunohistochemical analysis revealed a significant increase in Iba‐1^+^ microglia in the hippocampus of 3×Tg‐AD mice (Figure 4A,B). Consistent with this, Western blot results demonstrated upregulation of both Iba‐1 and GFAP (a marker of reactive astrocytes) in transgenic animals (Figure 4C–E). Notably, β‐asarone treatment markedly reduced the expression of these neuroinflammatory markers. Further supporting an anti‐inflammatory effect, ELISA assays indicated that β‐asarone significantly decreased hippocampal levels of IL‐1β, IL‐18, and TNF‐α. Together, these findings suggest that β‐asarone attenuates neuroinflammation in the 3×Tg‐AD mice.

### 3.5 β‐Asarone Treatment Inhibited NLRP3 Inflammasome Activation and Activated Autophagy in the Hippocampus of 3×Tg‐AD Mice

**FIGURE 5:**
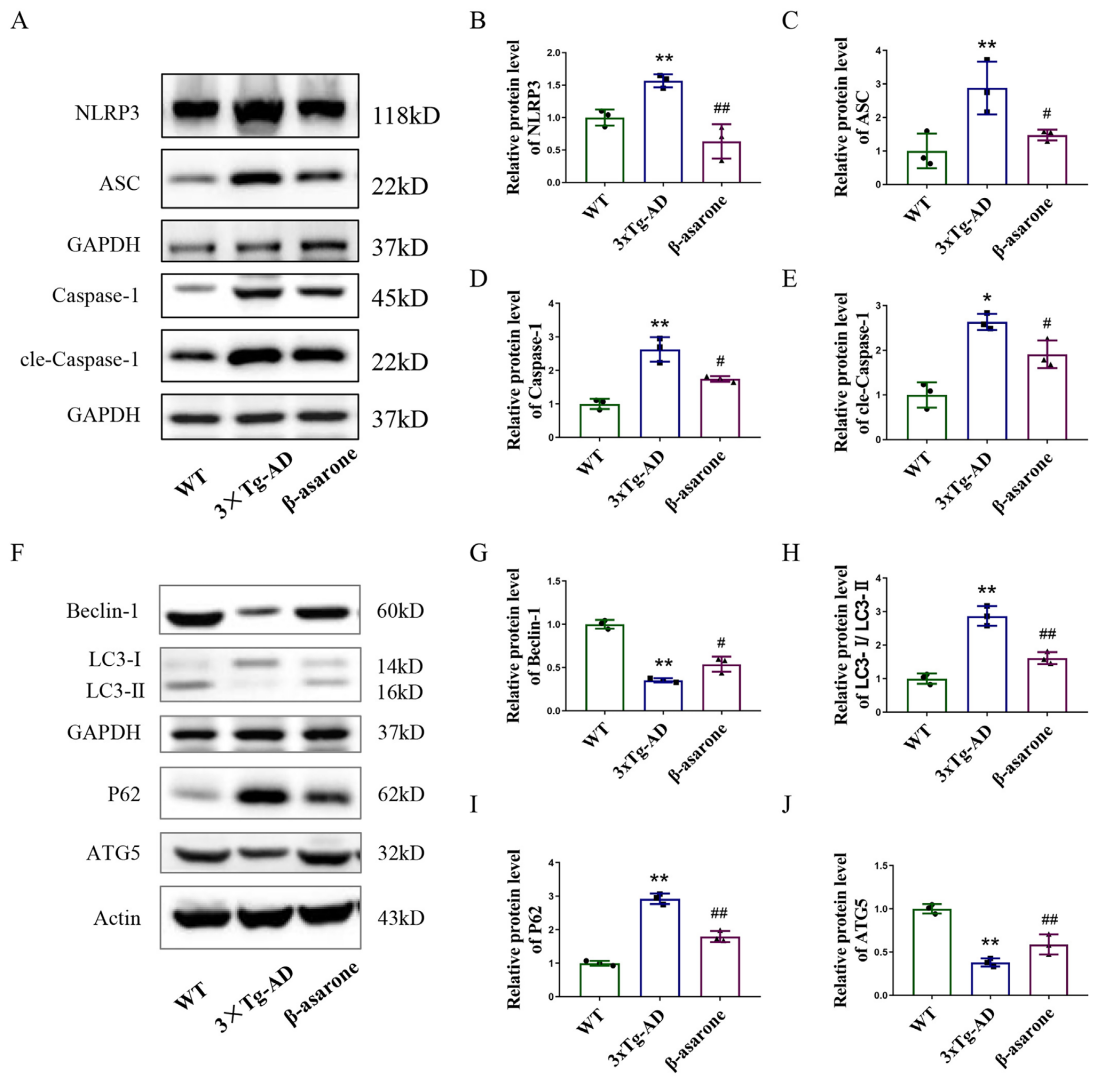
β‐asarone inhibited NLRP3 inflammasome and activated autophagy in the hippocampus of 3×Tg‐AD mice. (A) Representative immunoblots of NLRP3, ASC, Caspase‐1 and cle‐Caspase‐1. (B–E) Quantification of relative protein level of NLRP3, ASC, Caspase‐1 and cle‐Caspase‐1. (F) Representative immunoblots of Beclin‐1, LC2‐II/I, P62 and ATG5. (G–J) Quantification of relative protein level of Beclin‐1, LC2‐II/I, P62 and ATG5. Data are expressed as mean ± SD. Statistics: One‐way ANOVA with Tukey's test. *p < 0.05, **p < 0.01, relative to WT group; ^#^ *p* < 0.05, ^##^ *p* < 0.01, relative to 3×Tg‐AD group. *n* = 3/group.

Activation of the NLRP3 inflammasome results in the secretion of pro‐inflammatory cytokines such as IL‐1β and IL‐18. Conversely, the induction of autophagy serves to inhibit NLRP3 inflammasome activation, thereby mitigating excessive inflammatory responses. The outcomes of Western blot evaluations indicated significant elevations in the levels of NLRP3, ASC, and Caspase‐1 proteins associated with the NLRP3 inflammasome in 3×Tg‐AD mice, with all these markers showing considerable reductions following β‐asarone treatment (Figure 5A–E). In terms of autophagy‐related markers, P62 expression exhibited a significant increase, whereas the LC3‐II/I ratio, Beclin‐1, and ATG5 expressions substantially decreased in the 3×Tg‐AD mice. Notably, these alterations were reversible following β‐asarone treatment (Figure 5F–J). Therefore, the aforementioned indicators collectively affirm that β‐asarone inhibits NLRP3 inflammasome activation while promoting autophagy.

### 3.6 β‐Asarone Treatment Inhibited Microglia Apoptosis and Neuroinflammation Induced by Aβ

**FIGURE 6:**
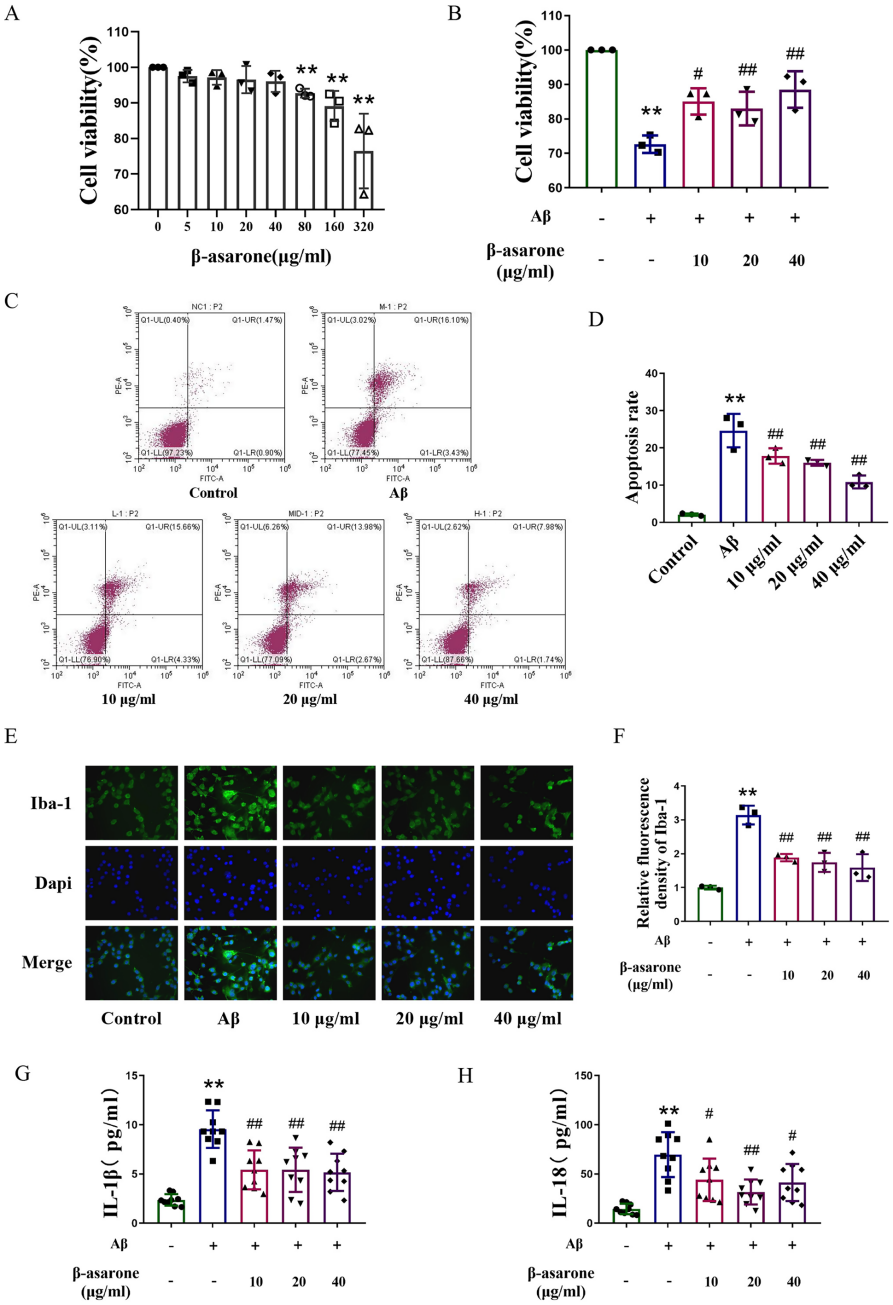
β‐asarone inhibited microglia apoptosis and neuroinflammation induced by Aβ. (A) The viability of normal BV‐2 cells with β‐asarone treatment(5–320 μg/mL, 24 h; CCK‐8 assay). (B) The viability of BV‐2 cells induced by Aβ_25–35_ with β‐asarone treatment. (C) Flow cytometry analysis of microglial apoptosis. Apoptosis was assessed by Annexin V‐FITC/PI double staining. Q1 (Annexin V^−^/PI^+^): Necrotic cells, Q2 (Annexin V^+^/PI^+^): Late apoptotic cells, Q3 (Annexin V^+^/PI^−^): Early apoptotic cells, Q4 (Annexin V^−^/PI^−^): Viable cells. (D) Apoptosis rate of microglia. Total apoptosis rate = Q2 + Q3. (E) Representative immunofluorescent images of Iba‐1 (green)/DAPI (blue) (400×). (F) Quantification of relative fluorescence density of Iba‐1. (G, H) ELISA of IL‐18 and IL‐1β in microglial supernatant. Data are expressed as mean ± SD. Statistics: One‐way ANOVA with Tukey's test. *p < 0.05, **p < 0.01, relative to contral group; ^#^ *p* < 0.05, ^##^ *p* < 0.01, relative to Aβ group. *n* = 3/group.

Microglia are the innate immune cells primarily responsible for the production of inflammatory cytokines. Hence, we focused our investigation on the effects of β‐asarone on microglia. Initially, we determined the appropriate dosage of β‐asarone. As depicted in Figure 6A,B, concentrations of β‐asarone below 80 μg/mL did not impact microglial activity, while concentrations exceeding 80 μg/mL significantly inhibited cellular activity. Simultaneously, 25 μM Aβ_25‐35_ was employed for cell culture to simulate the microglial environment associated with AD, revealing that doses of 10, 20, and 40 μg/mL of β‐asarone effectively modulated microglial responses.

The administration of β‐asarone at concentrations of 10, 20, and 40 μg/mL significantly enhanced cell viability, prompting us to select these specific dosages for subsequent cellular experiments. Flow cytometry was employed to assess the impact of β‐asarone on the apoptosis of microglial cells. The findings indicated that Aβ promotes microglial apoptosis, whereas β‐asarone effectively reduced the apoptosis rate in an Aβ‐rich environment (Figure 6C,D). Subsequently, we continued to investigate the role of β‐asarone in neuroinflammation through cellular assays. In an Aβ context, immunofluorescence analysis revealed a significant increase in Iba‐1 expression, which was notably decreased following β‐asarone treatment (Figure 6E,F). Additionally, ELISA results corroborated that inflammatory cytokines IL‐1β and IL‐18 levels in microglial supernatants were markedly elevated under Aβ conditions, with a subsequent decrease in these cytokines post β‐asarone intervention (Figure 6G,H). These results collectively demonstrate that β‐asarone administration mitigates neuroinflammation associated with microglial activation.

### 3.7 β‐Asarone Treatment Inhibited NLRP3 Inflammasome Activation and Activated Autophagy in Microglia Induced by Aβ

**FIGURE 7:**
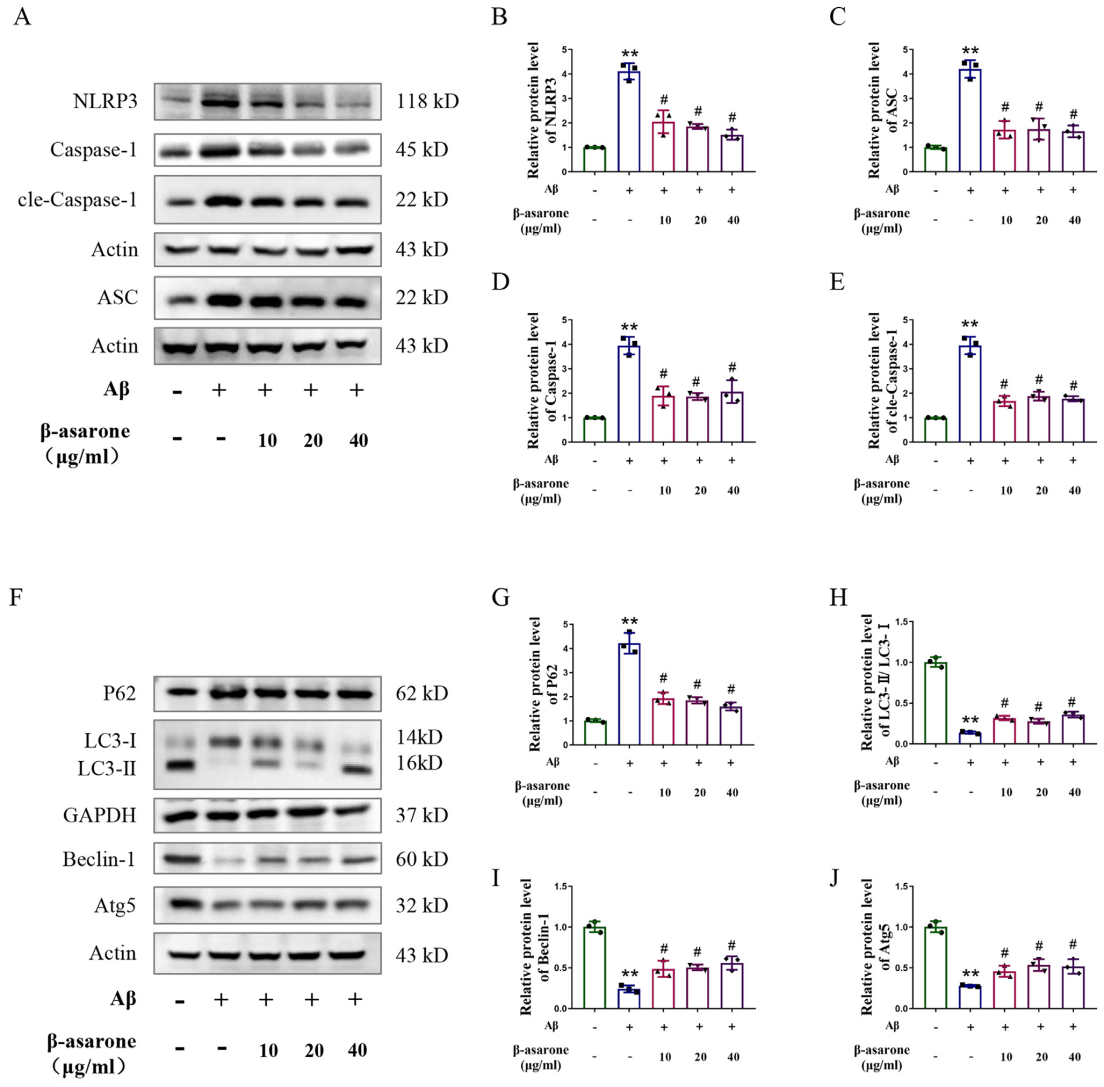
β‐asarone inhibited NLRP3 inflammasome and activated autophagy in microglia induced by Aβ. (A) Representative immunoblots of NLRP3, ASC, Caspase‐1 and cle‐Caspase‐1. (B–E) Quantification of relative protein level of NLRP3, ASC, Caspase‐1 and cle‐Caspase‐1. (F) Representative immunoblots of Beclin‐1, LC2‐II/I, P62 and ATG5. (G–J) Quantification of relative protein level of Beclin‐1, LC2‐II/I, P62 and ATG5. Data are expressed as mean ± SD. Statistics: One‐way ANOVA with Tukey's test and Kruskal–Wallis test *p < 0.05, **p < 0.01, relative to contral group; ^#^ *p* < 0.05, ^##^ *p* < 0.01, relative to Aβ group. *n* = 3/group.

Western blot was performed to detect the expressions of NLRP3 inflammasome and autophagy‐related proteins. The experimental results showed that the expressions of NLRP3, ASC, and Caspase‐1 proteins related to the NLRP3 inflammasome in the model group were significantly increased, and these indicators all went down significantly after treatment with β‐asarone (Figure 7A–E). As for autophagy‐related indicators Beclin‐1, LC3‐II/I ratio, P62, and ATG5, Beclin‐1 expression decreased significantly in the Aβ group, while the expressions of LC3‐II/I ratio, P62, and ATG5 increased significantly. The change trend of these indicators in the Aβ group could be reversed after treatment with β‐asarone (Figure 7F–J). Therefore, the above indicators confirmed that β‐asarone treatment inhibited NLRP3 inflammasome activation and activated autophagy in microglia.

### 3.8 Inhibition of Autophagy Attenuated the Inhibitory Effect of β‐Asarone on NLRP3 Inflammasome in Microglia Induced by Aβ

**FIGURE 8:**
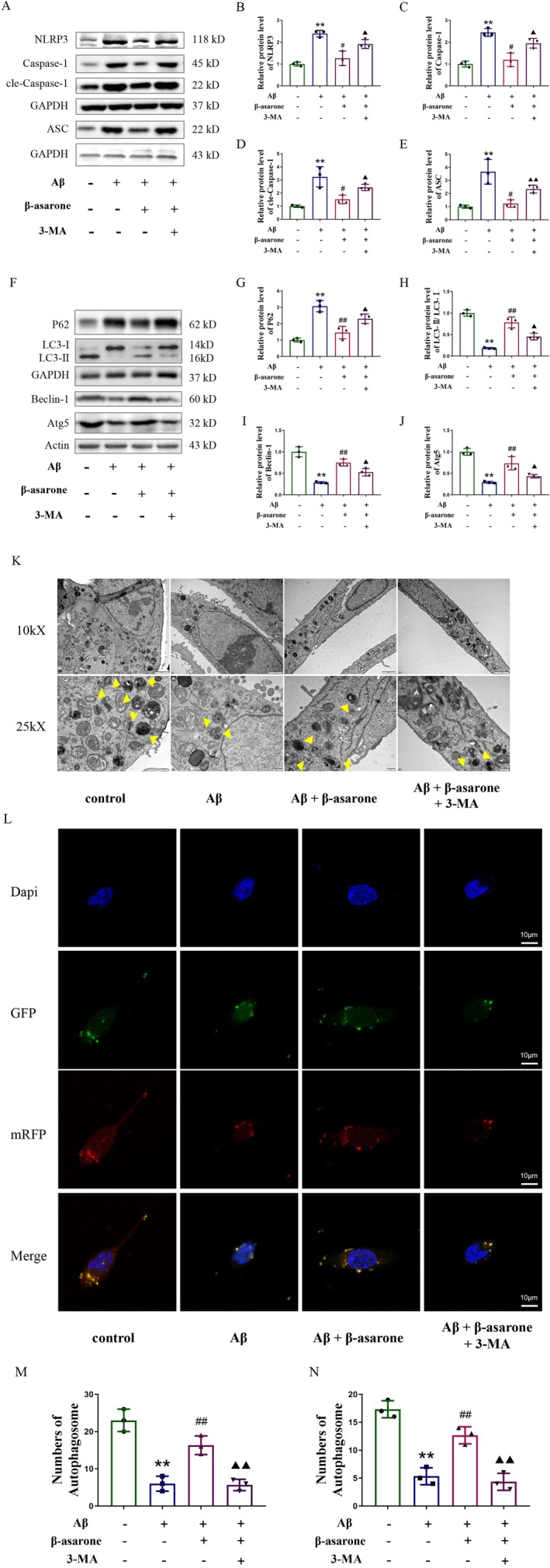
Inhibition of autophagy attenuated the inhibitory effect of β‐asarone on NLRP3 inflammasome in microglia induced by Aβ. (A) Representative immunoblots of NLRP3, ASC, Caspase‐1 and cle‐Caspase‐1. (B–E) Quantification of relative protein level of NLRP3, ASC, Caspase‐1 and cle‐Caspase‐1. (F) Representative immunoblots of Beclin‐1, LC3‐II/I, P62 and ATG5. (G–J) Quantification of relative protein level of Beclin‐1, LC3‐II/I, P62 and ATG5. (K) Representative TEM images showing autophagic vacuoles. 10kX: 2 μm; 25kX: 500 nm. (L) Autophagic flux monitored by mRFP‐GFP‐LC3 fluorescence. Representative merged (yellow/red) and single‐channel images show autophagic puncta. (M) Quantitative analysis of autophagosomes from TEM images. (N) Quantification of autophagic puncta from the mRFP‐GFP‐LC3 reporter assay. Data are expressed as mean ± SD. Statistics: One‐way ANOVA with Tukey's test. *p < 0.05, **p < 0.01, relative to contral group; ^#^ *p* < 0.05, ^##^ *p* < 0.01, relative to Aβ group. ^▲^ *p* < 0.05, ^▲▲^ *p* < 0.01, relative to β‐asarone group. *n* = 3/group. Aβ = 25 μM, β‐asarone = 40 μg/mL, 3‐MA = 10 μM.

To further confirm whether β‐asarone inhibited NLRP3 inflammasome by activating autophagy. BV2 cells were concurrently treated with β‐asarone and the autophagy inhibitor 3‐MA. The results showed that 3‐MA attenuated the inhibitory effect of β‐asarone on NLRP3, ASC, Caspase‐1, and cle‐Caspase‐1 (Figure 8A–E). Meanwhile, 3‐MA could block the increasing effect of β‐asarone on LC3‐II/I ratio, Beclin‐1, and Atg5, and block the decreasing effect of β‐asarone on P62 (Figure 8F–J). Both TEM and mRFP‐GFP‐LC3 adenovirus fluorescence assays demonstrated altered autophagic activity (Figure 8K–N). The model group exhibited a significant decrease in the number of autophagosomes. In contrast, drug treatment markedly increased autophagosome formation. This increase was effectively abolished by the autophagy inhibitor 3‐MA, which again significantly reduced autophagosome counts. Furthermore, we used the NLRP3 inhibitor MCC950 to verify whether it could affect the effect of β‐asarone on autophagy. The results showed that MCC950 was unable to inhibit the activation effect of β‐asarone on autophagy (Figure S1). Collectively, these experimental outcomes suggest that the inhibition of autophagy undermines the efficacy of β‐asarone against NLRP3 inflammasome activation in Aβ‐induced microglia, thereby indicating that β‐asarone exerts its effects through the activation of autophagy.

## 4 Discussion

The pathogenesis of AD remains uncertain, encompassing theories such as Aβ deposition, Tau protein hyperphosphorylation, cholinergic dysfunction, and excitatory amino acid toxicity. Among them, autophagy process disorders and neuroinflammation are also considered to be important causes [25]. β‐asarone has been shown to modulate multiple pathways implicated in Alzheimer's disease, including conferring neuroprotection, stimulating brain‐derived neurotrophic factor (BDNF) production and release, attenuating Aβ aggregation and plaque formation, promoting neurogenesis, and dampening neuroinflammatory responses [16, 17]. Some studies have shown that its mechanism of action is related to the activation of autophagy [26, 27]. Our current study focuses on whether β‐asarone can reduce the occurrence of neuroinflammation by activating the autophagy process.

In this experiment, we employed 3×Tg‐AD mice in this investigation. The mice were bred by combining APP_Swe_ and Tau_P301L_ genes into PS1_M146V_ mutant mice, and the constructed 3×Tg‐AD mice were able to form amyloid plaques, neurofibrilar tangles, and synaptic dysfunction in an age‐related manner. At present, it is the transgenic animal model that is closest to the pathological characteristics of AD [28]. MWM and Step‐down test are both commonly used behavioral methods to evaluate the mice cognitive performance [24]. To evaluate cognitive performance, the MWM and Step‐down Test were employed to assess the influence of β‐asarone on cognition in 3×Tg‐AD mice. The results indicated that β‐asarone administration significantly enhanced cognitive function in this AD model.

Research indicates that Alzheimer's disease patients experience substantial neuronal loss throughout the brain [29]. In this experiment, HE staining was used to observe the morphology of hippocampal CA3 and cortical neurons to determine the degree of neuron damage. The CA3 subregion of the hippocampus is crucial to the formation of spatial memory, which can support the formation of spatial arbitrary associations, the temporary maintenance of spatial working memory and the completion of spatial patterns [30]. Therefore, we choose CA3 as the object of observation. Nishi bodies are widely distributed in neuronal cell bodies or dendrites, and their main function is to synthesize various proteins required by cells, including structural proteins, neurotransmitters and various enzymes. The number of Nissl bodies can reflect the functional state of neurons [31]. Neuronal nuclei (NeuN) are well‐established markers detected only in postmitotic neurons and stably expressed at specific developmental stages, and are considered reliable biomarkers of mature neurons [32, 33]. In the present experiment, after the intervention of β‐asarone, the nuclear pyknosis, cytoplasmic eosinophilia and structural integrity of the hippocampal neurons have all been significantly improved, the number of Nishi bodies has increased, which indicated that the damaged neurons had been improved. Meanwhile, Western Blot and immunofluorescence were used to detect the expression of NeuN and PSD95 in the hippocampus of 3×Tg‐AD mice. The results showed that the expression of NeuN and PSD95 in the hippocampus of 3×Tg‐AD mice was decreased, while β‐asarone could increase the expression of NeuN in the hippocampus of 3×TG‐AD mice. Reduced NeuN and PSD95 independently confirmed neuronal loss, supporting HE/Nissl findings. Consistent with this, Fluoro‐Jade B (FJB) staining provided further evidence of neuronal degeneration. Therefore, the above experimental results confirm that β‐asarone can improve the pathological injury and neuronal function of 3×Tg‐AD mice.

The main pathological features of AD are extracellular Aβ deposition and intracellular Tau protein hyperphosphorylation. Aβ is formed from a single transmembrane β‐amyloid precursor protein (APP) cleaved by β‐secretase and γ‐secretase. The cleaved Aβ mostly contains 27–43 amino acids and is deposited by cytoendocrine to intercellular polymerization [34]. Tau proteins need to maintain a balance between phosphorylation and dephosphorylation to maintain the normal physiological function of neurons. Abnormal phosphorylation of Tau leads to decreased microtubule binding affinity, inhibition of glutamate receptor transport, or synaptic anchoring, which impels synaptic function [35]. In this experiment, after β‐asarone intervention, the expression of Aβ and phosphorylated Tau Ser396 were significantly reduced, indicating that β‐asarone could alleviate the characteristic protein changes of AD.

Recent studies have shown that neuroinflammation mediated by microglial (MG) is at the center of the pathogenesis of AD, and MG is the main immune cell in the central inflammatory response. Early activated MG is conducive to clearing toxic Aβ from the brain, but with the development of AD, MG phagocytosis activity decreases, excessive Aβ deposition produces more pro‐inflammatory factors and neurotoxic substances, thus accelerating AD progression. That is, early inflammation is beneficial, and long‐term inflammation can lead to further exacerbation of AD [36]. Iba‐1, which is stable and abundant in microglia, is a specific marker for microglia and has been widely used for microglia detection [37]. It has been extensively reported that activated microglia in the central nervous system lead to upregulation of Iba‐1 [38]. GFAP is the main intermediate filament protein in mature astrocytes, and it is the hallmark of astrocyte development. Increased GFAP expression is a sign of active reactive gliosis, and therefore, GFAP is a marker of astrocyte activation, which causes changes in GFAP in almost all neurological diseases [39]. Inflammatory cytokines in the central nervous system are usually produced by microglia, and the common inflammatory cytokines involved in the inflammatory response include IL‐1β, TNF‐α, IL‐18, IL‐6, etc., [40]. Therefore, in this experiment, we chose to detect Iba‐1, GFAP and inflammatory factors IL‐1β, TNF‐α and IL‐18 indicators to reflect the central nervous system inflammation. In the animal experiments, the results showed that β‐asarone could reduce the expression of Iba‐1 and GFAP in the hippocampal tissue of 3×Tg‐AD mice, and reduce the levels of inflammatory cytokines IL‐1β, TNF‐α and IL‐18. Similarly, in the cell experiments, after β‐asarone treatment, the expression of Iba‐1 in BV‐2 microglia induced by Aβ was decreased, and the contents of IL‐1β and IL‐18 were also significantly decreased. Therefore, the above findings confirm that β‐asarone can reduce neuroinflammation caused by microglia.

IL‐1β and IL‐18 are the characteristic inflammatory factors of NLRP3 inflammasome‐mediated inflammatory response, which includes initiation and activation phases. In the initiation phase, toll‐like receptors such as TLR on MG membrane recognize the corresponding ligand when stimulated by signals, which can activate the NF‐κB pathway and promote the formation of NLRP3, Caspase‐1, ASC, pro‐IL‐1β, pro‐IL‐18 and other precursor proteins. In the activation stage, NLRP3, ASC, and pro‐caspase‐1 combine to form an active NLRP3 inflammasome and activate Caspase‐1 under the stimulation of external conditions such as ion channel dysregulation, lysosome injury, and oxidative stress. Caspase‐1 can hydrolyze pro‐IL‐1β and pro‐IL‐18 to mature IL‐1β and IL‐18 [41, 42, 43]. The results showed that the NLRP3 inflammator‐related proteins NLRP3, ASC, Caspase‐1 and pro‐caspase‐1 in the hippocampus of 3×Tg‐AD mice decreased after β‐asarone treatment, as did the results in the cell experiments. These results suggest that β‐asarone can inhibit neuroinflammation mediated by the NLRP3 inflammasome.

Autophagy is an essential cellular quality‐control process that maintains homeostasis through lysosome‐mediated degradation of sequestered cargo—including misfolded proteins and impaired mitochondria—within autophagosomes [44]. Eukaryotic cells respond to external stimuli through mechanisms linked to both the innate and adaptive immune systems, closely associating with the NLRP3 inflammasome. Inflammasomes and autophagy engage in reciprocal regulation critical for immune balance. Autophagy suppresses inflammasome activation by clearing activators like damaged mitochondria and degrading components. Conversely, inflammasome signaling can inhibit autophagy by disrupting lysosomal function and cleaving autophagy proteins. This dynamic extends beyond simple inhibition: autophagy supports inflammasome priming through NF‐κB, while inflammasomes can promote targeted autophagy. Dysregulation contributes to disease; defective autophagy exacerbates inflammation in metabolic disorders, while excessive autophagy may impair infection control. Therapeutically targeting this crosstalk (e.g., boosting autophagy to dampen NLRP3) holds significant promise [45, 46]. As mentioned above, β‐asarone can activate autophagy, so we also detected autophagy related proteins Beclin‐1, P62, ATG5 and LC‐II/I, and the results also confirmed that β‐asarone can not only inhibit the NLRP3 inflammasome‐mediated inflammatory response, but also promote autophagy. Consistently, assessments using TEM and the mRFP‐GFP‐LC3 reporter revealed that β‐asarone increased the number of autophagosomes. To further investigate whether β‐asarone inhibits NLRP3 inflammasome formation by promoting autophagy, we treated cells with the autophagy inhibitor 3‐MA and NLRP3 inhibitor MCC950. The results demonstrated that β‐asarone failed to attenuate NLRP3 inflammasome‐mediated inflammation following 3‐MA co‐treatment, whereas MCC950 co‐treatment did not affect β‐asarone‐induced autophagy, collectively confirming that β‐asarone alleviates NLRP3 inflammasome‐driven inflammatory responses specifically through autophagy activation.

## 5 Conclusion

In conclusion, our findings indicate that β‐asarone provides neuroprotection against neuroinflammation in 3×Tg‐AD mice by inhibiting the NLRP3 inflammasome through the activation of autophagy.

## Author Contributions

Zhiwei Xu, Wanying Xu, Jinxin He, and Jiahui Qian designed the study, analyzed and interpreted the data, and wrote the manuscript. Changyu Li and Xiaojie Zhou designed the study and revised the manuscript. All authors have read and agreed to the published version of the manuscript.

## Funding

Central Zhejiang Science and Technology Innovation Corridor Joint Fund of Zhejiang Provincial Natural Science Foundation of China, LJHSQY26H280002; Jinhua Traditional Chinese medicine science and technology project, 2025CC02; Zhejiang Province Traditional Chinese medicine science and technology project, 2025ZL061; Jinhua major key science and technology plan project, 2024‐3‐094.

## Ethics Statement

The study received approval from the Ethics and Laboratory Animal Management Committee at Zhejiang Chinese Medical University (Approval No. 20220607).

## Conflicts of Interest

The authors declare no conflicts of interest.

## Supporting information


**Figure S1:** Inhibition of NLRP3 ineffectual the activation of β‐asarone on autophagy in microglia induced by Aβ. (A) Representative immunoblots of NLRP3, Beclin‐1 and LC3‐II/I. (B–D) Quantification of relative protein level of NLRP3, Beclin‐1 and LC3‐II/I. Data are expressed as mean ± SD. *p < 0.05, **p < 0.01, relative to contral group; ^#^ *p* < 0.05, ^##^ *p* < 0.01, relative to Aβ group. *n* = 3. Aβ = 25 μM, β‐asarone = 40 μg/mL, MCC950 = 10 μM.

## Data Availability

The datasets utilized or examined in this study can be obtained from the corresponding author upon reasonable request. Detailed blot images can be found in Supporting Information.

## References

[1] M. Jucker and L. C. Walker , “Alzheimer's Disease: From Immunotherapy to Immunoprevention,” Cell 186, no. 20 (2023): 4260–4270. 37729908 10.1016/j.cell.2023.08.021 PMC10578497

[2] D. S. Knopman , H. Amieva , R. C. Petersen , et al., “Alzheimer Disease,” Nature Reviews. Disease Primers 7, no. 1 (2021): 33. 10.1038/s41572-021-00269-y PMC8574196 33986301

[3] J. M. Long and D. M. Holtzman , “Alzheimer Disease: An Update on Pathobiology and Treatment Strategies,” Cell 179, no. 2 (2019): 312–339. 31564456 10.1016/j.cell.2019.09.001 PMC6778042

[4] T. Athar , K. Al Balushi , and S. A. Khan , “Recent Advances on Drug Development and Emerging Therapeutic Agents for Alzheimer's Disease,” Molecular Biology Reports 48, no. 7 (2021): 5629–5645. 34181171 10.1007/s11033-021-06512-9 PMC8236749

[5] E. Liu , Y. Zhang , and J. Z. Wang , “Updates in Alzheimer's Disease: From Basic Research to Diagnosis and Therapies,” Translational Neurodegeneration 13, no. 1 (2024): 45. 39232848 10.1186/s40035-024-00432-x PMC11373277

[6] B. Twarowski and M. Herbet , “Inflammatory Processes in Alzheimer's Disease‐Pathomechanism, Diagnosis and Treatment: A Review,” International Journal of Molecular Sciences 24, no. 7 (2023): 6518. 37047492 10.3390/ijms24076518 PMC10095343

[7] Y. Cai , J. Liu , B. Wang , M. Sun , and H. Yang , “Microglia in the Neuroinflammatory Pathogenesis of Alzheimer's Disease and Related Therapeutic Targets,” Frontiers in Immunology 13 (2022): 856376. 35558075 10.3389/fimmu.2022.856376 PMC9086828

[8] R. Lu , L. Zhang , and X. Yang , “Interaction Between Autophagy and the NLRP3 Inflammasome in Alzheimer's and Parkinson's Disease,” Frontiers in Aging Neuroscience 14 (2022): 1018848. 36262883 10.3389/fnagi.2022.1018848 PMC9574200

[9] D. Zhang , Y. Zhang , J. Pan , et al., “Degradation of NLRP3 by p62‐Dependent‐Autophagy Improves Cognitive Function in Alzheimer's Disease by Maintaining the Phagocytic Function of Microglia,” CNS Neuroscience & Therapeutics 29, no. 10 (2023): 2826–2842. 37072933 10.1111/cns.14219 PMC10493665

[10] C. Deng , H. Chen , Z. Meng , and S. Meng , “Roles of Traditional Chinese Medicine Regulating Neuroendocrinology on AD Treatment,” Frontiers in Endocrinology 13 (2022): 955618. 36213283 10.3389/fendo.2022.955618 PMC9533021

[11] W. Tan , L. Qi , X. Hu , and Z. Tan , “Research Progress in Traditional Chinese Medicine in the Treatment of Alzheimer's Disease and Related Dementias,” Frontiers in Pharmacology 13 (2022): 921794. 36506569 10.3389/fphar.2022.921794 PMC9729772

[12] M. R. Ding , Y. J. Qu , B. Hu , and H. M. An , “Signal Pathways in the Treatment of Alzheimer's Disease With Traditional Chinese Medicine,” Biomedicine & Pharmacotherapy 152 (2022): 113208. 35660246 10.1016/j.biopha.2022.113208

[13] M. Wang , H. P. Tang , S. Wang , et al., “Acorus Tatarinowii Schott: A Review of Its Botany, Traditional Uses, Phytochemistry, and Pharmacology,” Molecules 28, no. 11 (2023): 4525. 37299001 10.3390/molecules28114525 PMC10254448

[14] Z. Xu , X. Zhou , X. Hong , et al., “Essential Oil of Acorus Tatarinowii Schott Inhibits Neuroinflammation by Suppressing NLRP3 Inflammasome Activation in 3×Tg‐AD Transgenic Mice,” Phytomedicine 112 (2023): 154695. 36774844 10.1016/j.phymed.2023.154695

[15] J. Huang , Z. Xu , C. Yu , et al., “The Volatile Oil of Acorus Tatarinowii Schott Ameliorates Alzheimer's Disease Through Improving Insulin Resistance via Activating the PI3K/AKT Pathway,” Phytomedicine 135 (2024): 156168. 39486109 10.1016/j.phymed.2024.156168

[16] X. Y. Du , Y. S. Cao , J. Yang , et al., “Preclinical Evidence and Possible Mechanisms of β‐Asarone for Rats and Mice With Alzheimer's Disease: A Systematic Review and Meta‐Analysis,” Frontiers in Pharmacology 13 (2022): 956746. 36120381 10.3389/fphar.2022.956746 PMC9471869

[17] R. Balakrishnan , D. Y. Cho , I. S. Kim , S. H. Seol , and D. K. Choi , “Molecular Mechanisms and Therapeutic Potential of α‐ and β‐Asarone in the Treatment of Neurological Disorders,” Antioxidants (Basel) 11, no. 2 (2022): 281. 35204164 10.3390/antiox11020281 PMC8868500

[18] H. B. Guo , Y. F. Cheng , J. G. Wu , et al., “Donepezil Improves Learning and Memory Deficits in APP/PS1 Mice by Inhibition of Microglial Activation,” Neuroscience 290 (2015): 530–542. 25662507 10.1016/j.neuroscience.2015.01.058

[19] L. H. Li , W. N. Peng , Y. Deng , J. J. Li , and X. R. Tian , “Action of Trichostatin A on Alzheimer's Disease‐Like Pathological Changes in SH‐SY5Y Neuroblastoma Cells,” Neural Regeneration Research 15, no. 2 (2020): 293–301. 31552902 10.4103/1673-5374.265564 PMC6905323

[20] X. Zhu , J. Gao , and C. Qiu , “Integrative Analysis Reveals Key Lysosomal Genes as Potential Therapeutic Targets in Alzheimer's Disease,” Metabolic Brain Disease 39, no. 7 (2024): 1433–1445. 39150655 10.1007/s11011-024-01409-5 PMC11513730

[21] N. Shao , Z. Ding , F. Liu , et al., “Huang‐Pu‐Tong‐Qiao Formula Alleviates Hippocampal Neuron Damage by Inhibiting NLRP3 Inflammasome‐Mediated Pyroptosis in Alzheimer's Disease,” Molecular Neurobiology 62 (2024): 2456. 10.1007/s12035-024-04547-0 39466576

[22] P. Xu , Z. Li , H. Wang , X. Zhang , and Z. Yang , “Triptolide Inhibited Cytotoxicity of Differentiated PC12 Cells Induced by Amyloid‐Beta_25–35_ via the Autophagy Pathway,” PLoS One 10, no. 11 (2015): e0142719. 26554937 10.1371/journal.pone.0142719 PMC4640509

[23] Y. Luo , T. Ye , H. Tian , et al., “Empagliflozin Alleviates Obesity‐Related Cardiac Dysfunction via the Activation of SIRT3‐Mediated Autophagosome Formation,” Lipids in Health and Disease 23, no. 1 (2024): 308. 39334359 10.1186/s12944-024-02293-9 PMC11430456

[24] N. S. Pentkowski , K. K. Rogge‐Obando , T. N. Donaldson , S. J. Bouquin , and B. J. Clark , “Anxiety and Alzheimer's Disease: Behavioral Analysis and Neural Basis in Rodent Models of Alzheimer's‐Related Neuropathology,” Neuroscience and Biobehavioral Reviews 127 (2021): 647–658. 33979573 10.1016/j.neubiorev.2021.05.005 PMC8292229

[25] P. Ou‐Yang , Z. Y. Cai , and Z. H. Zhang , “Molecular Regulation Mechanism of Microglial Autophagy in the Pathology of Alzheimer's Disease,” Aging and Disease 14, no. 4 (2023): 1166–1177. 37163443 10.14336/AD.2023.0106 PMC10389815

[26] M. Deng , L. Huang , and X. Zhong , “β‑Asarone Modulates Beclin‑1, LC3 and p62 Expression to Attenuate Aβ40 and Aβ42 Levels in APP/PS1 Transgenic Mice With Alzheimer's Disease,” Molecular Medicine Reports 21, no. 5 (2020): 2095–2102. 32186763 10.3892/mmr.2020.11026 PMC7115210

[27] N. Wang , H. Wang , L. Li , Y. Li , and R. Zhang , “β‐Asarone Inhibits Amyloid‐β by Promoting Autophagy in a Cell Model of Alzheimer's Disease,” Frontiers in Pharmacology 10 (2019): 1529. 32009952 10.3389/fphar.2019.01529 PMC6979317

[28] H. Sasaguri , S. Hashimoto , N. Watamura , et al., “Recent Advances in the Modeling of Alzheimer's Disease,” Frontiers in Neuroscience 16 (2022): 807473. 35431779 10.3389/fnins.2022.807473 PMC9009508

[29] G. He , Y. Li , H. Deng , and H. Zuo , “Advances in the Study of Cholinergic Circuits in the Central Nervous System,” Annals of Clinical Translational Neurology 10, no. 12 (2023): 2179–2191. 37846148 10.1002/acn3.51920 PMC10723250

[30] P. E. Gilbert and A. M. Brushfield , “The Role of the CA3 Hippocampal Subregion in Spatial Memory: A Process Oriented Behavioral Assessment,” Progress in Neuro‐Psychopharmacology & Biological Psychiatry 33, no. 5 (2009): 774–781. 19375477 10.1016/j.pnpbp.2009.03.037 PMC2743458

[31] J. Niu , C. Li , H. Wu , et al., “Propidium Iodide (PI) Stains Nissl Bodies and May Serve as a Quick Marker for Total Neuronal Cell Count,” Acta Histochemica 117, no. 2 (2015): 182–187. 25596876 10.1016/j.acthis.2014.12.001

[32] W. Duan , Y. P. Zhang , Z. Hou , et al., “Novel Insights Into NeuN: From Neuronal Marker to Splicing Regulator,” Molecular Neurobiology 53, no. 3 (2016): 1637–1647. 25680637 10.1007/s12035-015-9122-5

[33] V. V. Gusel'nikova and D. E. Korzhevskiy , “NeuN as a Neuronal Nuclear Antigen and Neuron Differentiation Marker,” Acta Naturae 7, no. 2 (2015): 42–47. 26085943 PMC4463411

[34] E. Fedele , “Anti‐Amyloid Therapies for Alzheimer's Disease and the Amyloid Cascade Hypothesis,” International Journal of Molecular Sciences 24, no. 19 (2023): 14499. 37833948 10.3390/ijms241914499 PMC10578107

[35] N. Younas , T. Saleem , A. Younas , and I. Zerr , “Nuclear Face of Tau: An Inside Player in Neurodegeneration,” Acta Neuropathologica Communications 11, no. 1 (2023): 196. 38087392 10.1186/s40478-023-01702-x PMC10714511

[36] N. Sun , M. B. Victor , Y. P. Park , et al., “Human Microglial State Dynamics in Alzheimer's Disease Progression,” Cell 186, no. 20 (2023): 4386–4429. 37774678 10.1016/j.cell.2023.08.037 PMC10644954

[37] J. Tischer , M. Krueger , W. Mueller , et al., “Inhomogeneous Distribution of Iba‐1 Characterizes Microglial Pathology in Alzheimer's Disease,” Glia 64, no. 9 (2016): 1562–1572. 27404378 10.1002/glia.23024

[38] Q. A. Xu , P. Boerkoel , V. Hirsch‐Reinshagen , et al., “Müller Cell Degeneration and Microglial Dysfunction in the Alzheimer's Retina,” Acta Neuropathologica Communications 10, no. 1 (2022): 145. 36199154 10.1186/s40478-022-01448-y PMC9533552

[39] M. Brenner and A. Messing , “Regulation of GFAP Expression,” ASN Neuro 13 (2021): 81206. 10.1177/1759091420981206 PMC7897836 33601918

[40] F. Zipp , S. Bittner , and D. P. Schafer , “Cytokines as Emerging Regulators of Central Nervous System Synapses,” Immunity 56, no. 5 (2023): 914–925. 37163992 10.1016/j.immuni.2023.04.011 PMC10233069

[41] P. Yu , X. Zhang , N. Liu , L. Tang , C. Peng , and X. Chen , “Pyroptosis: Mechanisms and Diseases,” Signal Transduction and Targeted Therapy 6, no. 1 (2021): 128. 33776057 10.1038/s41392-021-00507-5 PMC8005494

[42] Z. Rao , Y. Zhu , P. Yang , et al., “Pyroptosis in Inflammatory Diseases and Cancer,” Theranostics 12, no. 9 (2022): 4310–4329. 35673561 10.7150/thno.71086 PMC9169370

[43] S. O. Vasudevan , B. Behl , and V. A. Rathinam , “Pyroptosis‐Induced Inflammation and Tissue Damage,” Seminars in Immunology 69 (2023): 101781. 37352727 10.1016/j.smim.2023.101781 PMC10598759

[44] S. Liu , S. Yao , H. Yang , S. Liu , and Y. Wang , “Autophagy: Regulator of Cell Death,” Cell Death & Disease 14, no. 10 (2023): 648. 37794028 10.1038/s41419-023-06154-8 PMC10551038

[45] Z. Cao , Y. Wang , Z. Long , and G. He , “Interaction Between Autophagy and the NLRP3 Inflammasome,” Acta Biochimica et Biophysica Sinica Shanghai 51, no. 11 (2019): 1087–1095. 10.1093/abbs/gmz098 31609412

[46] A. G. Wu , X. G. Zhou , G. Qiao , et al., “Targeting Microglial Autophagic Degradation in NLRP3 Inflammasome‐Mediated Neurodegenerative Diseases,” Ageing Research Reviews 65 (2021): 101202. 33161129 10.1016/j.arr.2020.101202

