# Supplementary material for: β‐Asarone Attenuates Neuroinflammation of Alzheimer's Disease by Activating Autophagy and Suppressing NLRP3 Inflammasome Assembly
Source: CNS Neurosci Ther. 2026 Feb 4;32(2):e70771. doi: 10.1002/cns.70771 (PMC12869116; doi:10.1002/cns.70771)
Supplement: Supplementary file 1 — Figure S1: Inhibition of NLRP3 ineffectual the activation of β‐asarone on autophagy in microglia induced by Aβ. (A) Representative immunoblots of NLRP3, Beclin‐1 and LC3‐II/I. (B–D) Quantification of relative protein level of NLRP3, Beclin‐1 and LC3‐II/I. Data are expressed as mean ± SD. *p < 0.05, **p < 0.01, relative to contral group; # p < 0.05, ## p < 0.01, relative to Aβ group. n = 3. Aβ = 25 μM, β‐asarone = 40 μg/mL, MCC950 = 10 μM. [file CNS-32-e70771-s001.zip › cns70771-sup-0002-Supinfo.pdf]

Uncropped image of each cropped gel/blot

The lanes shown in Figures are marked with red boxes.

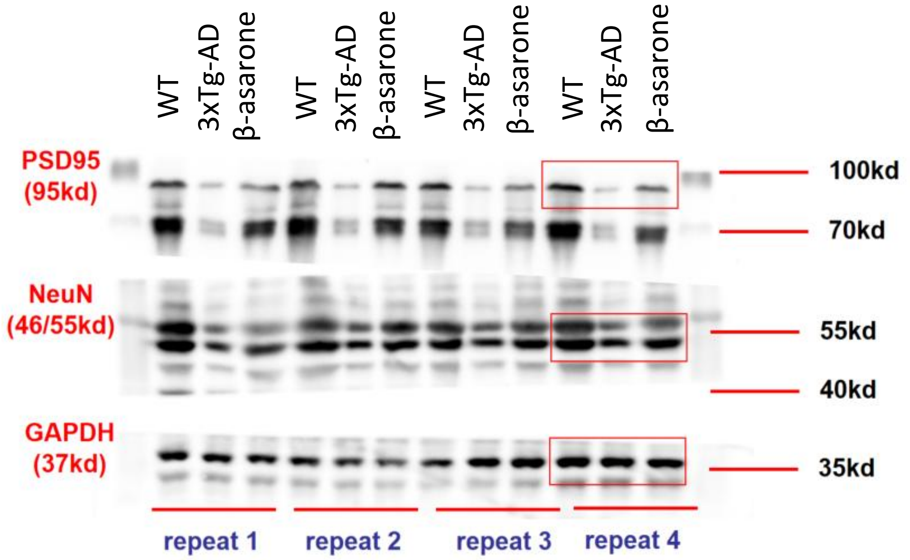

Full unedited gel/blot for Figure 2. G

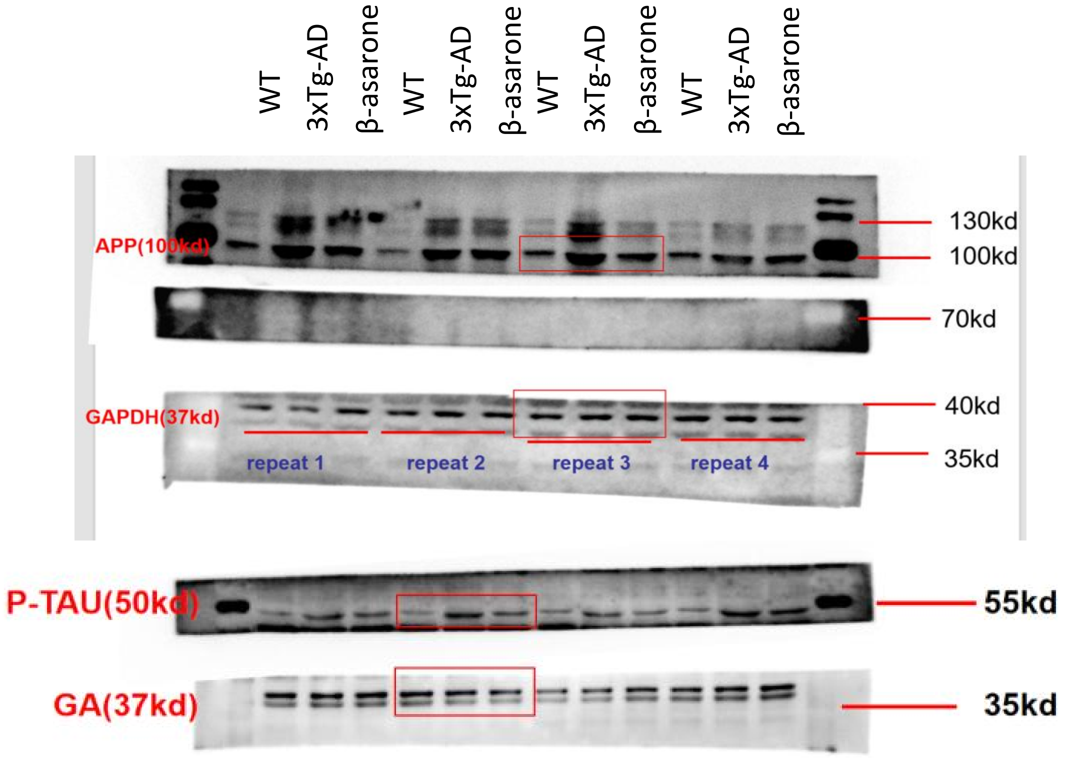

Full unedited gel/blot for Figure 3. C

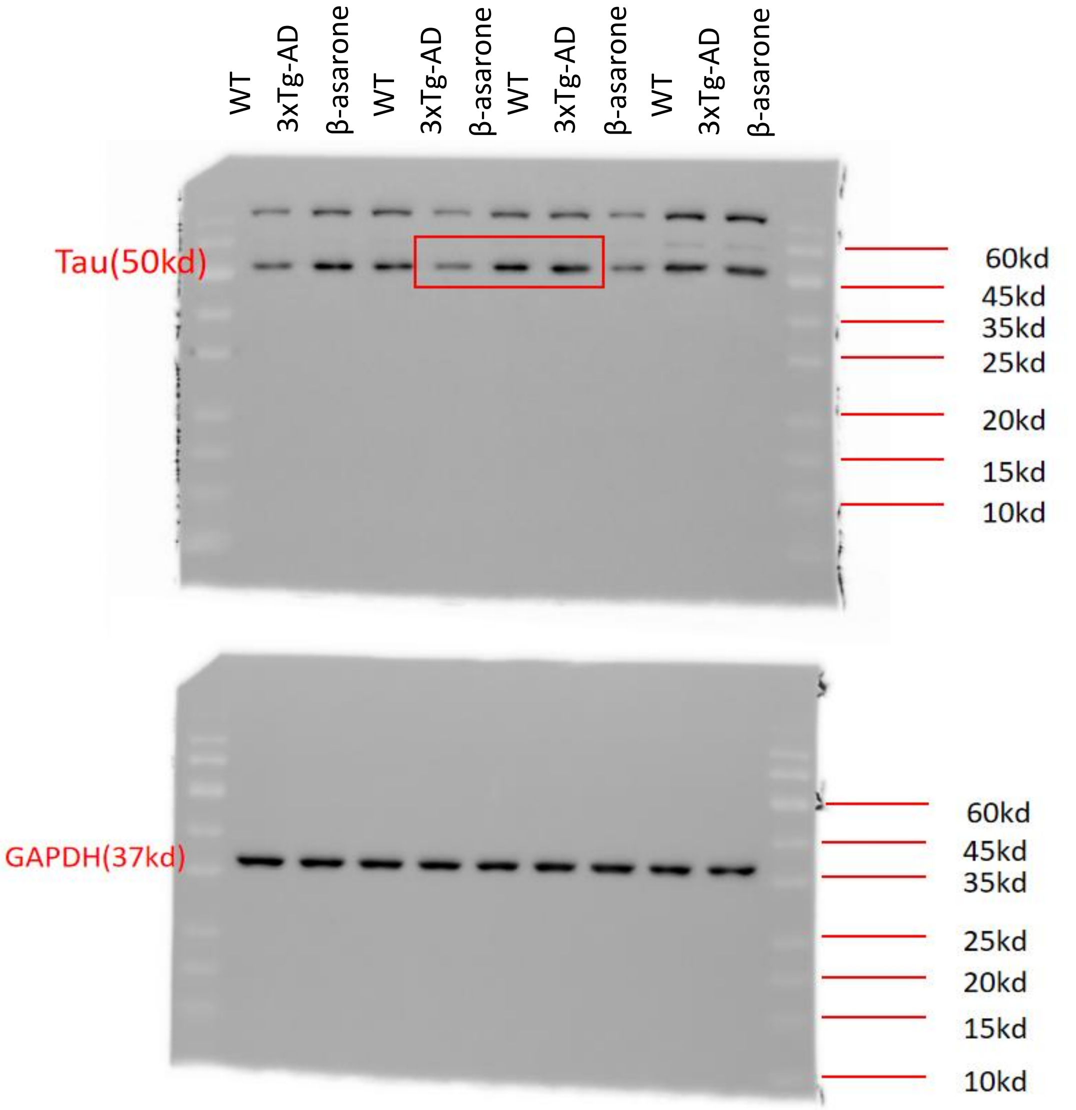

Full unedited gel/blot for Figure 3. C

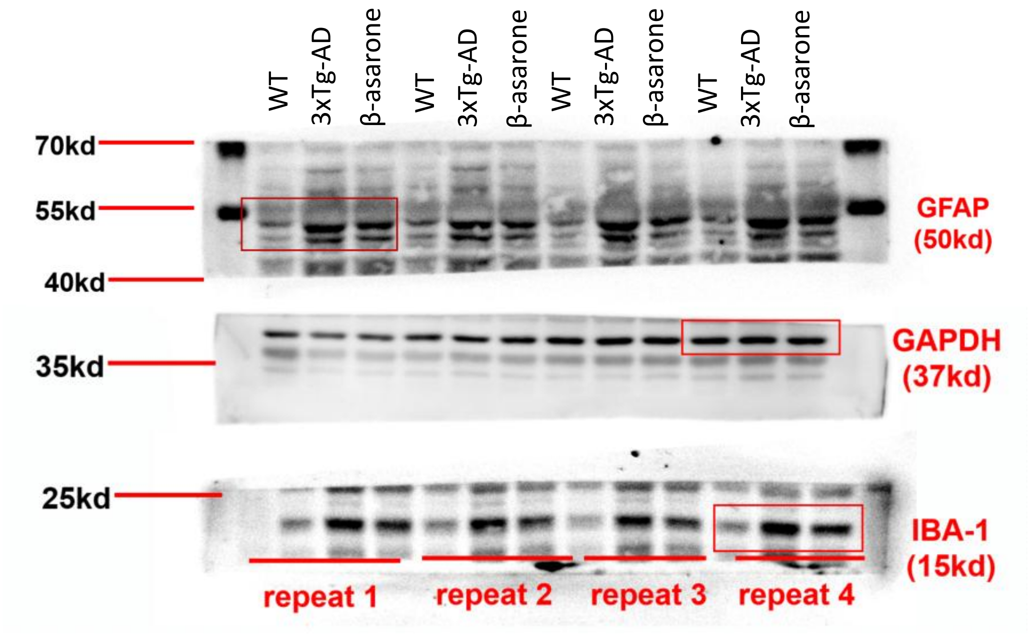

Full unedited gel/blot for Figure 4. C

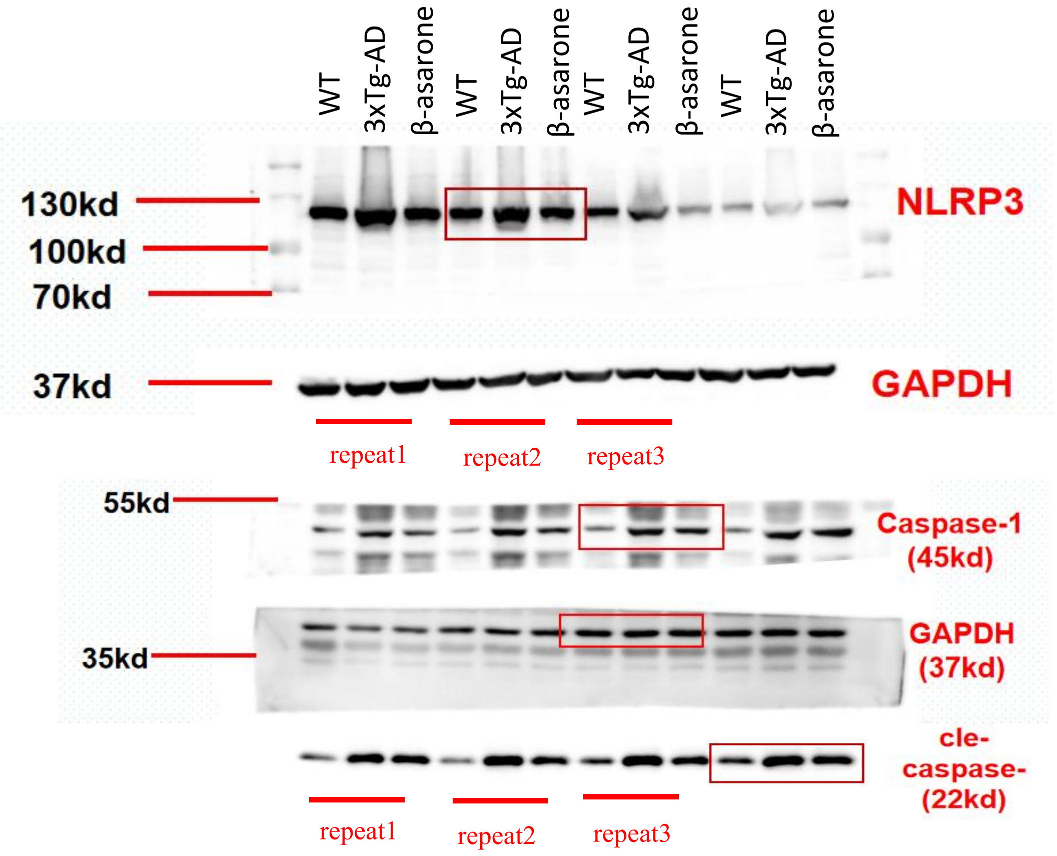

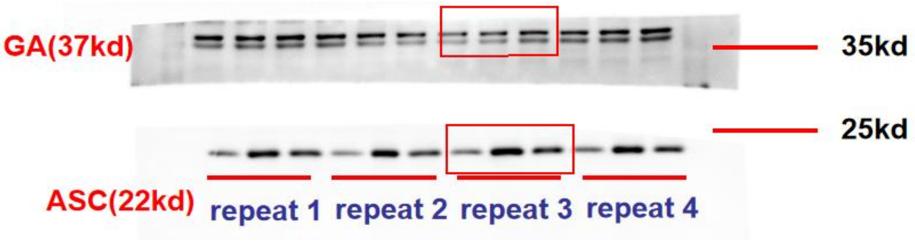

Full unedited gel/blot for Figure 5. A

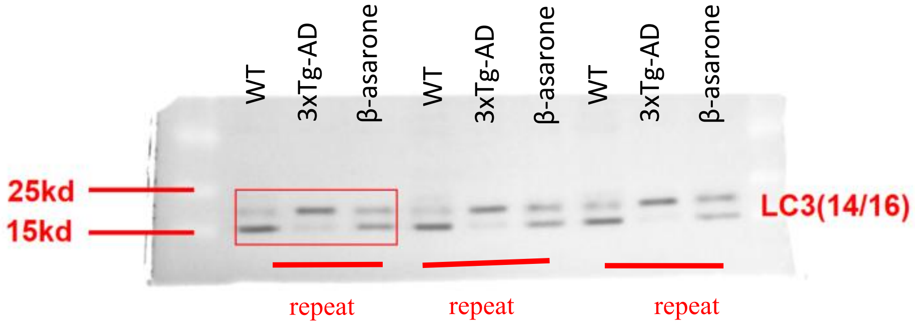

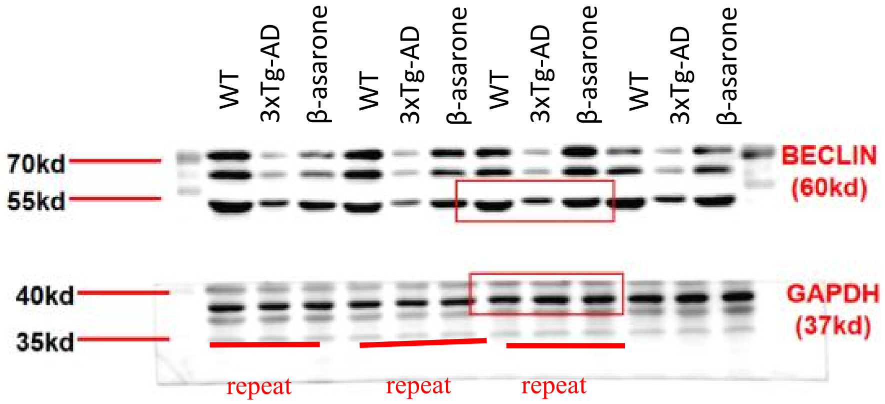

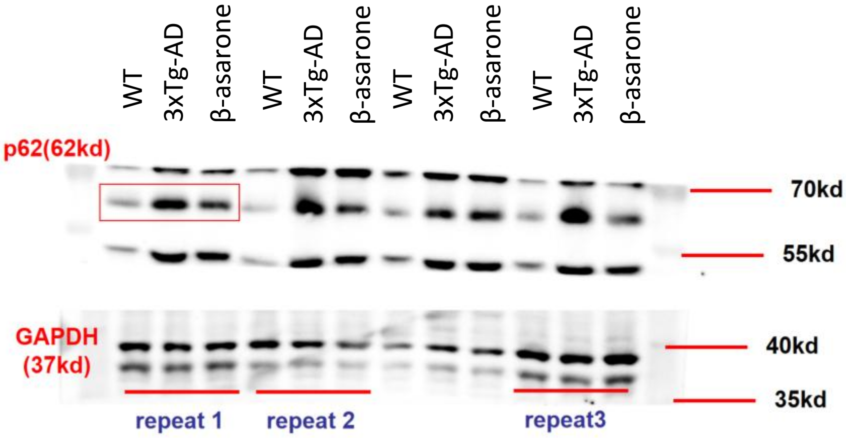

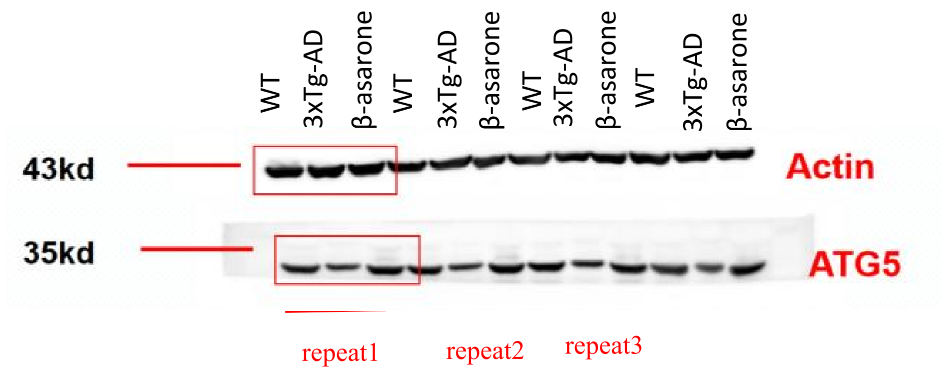

Full unedited gel/blot for Figure 5. F

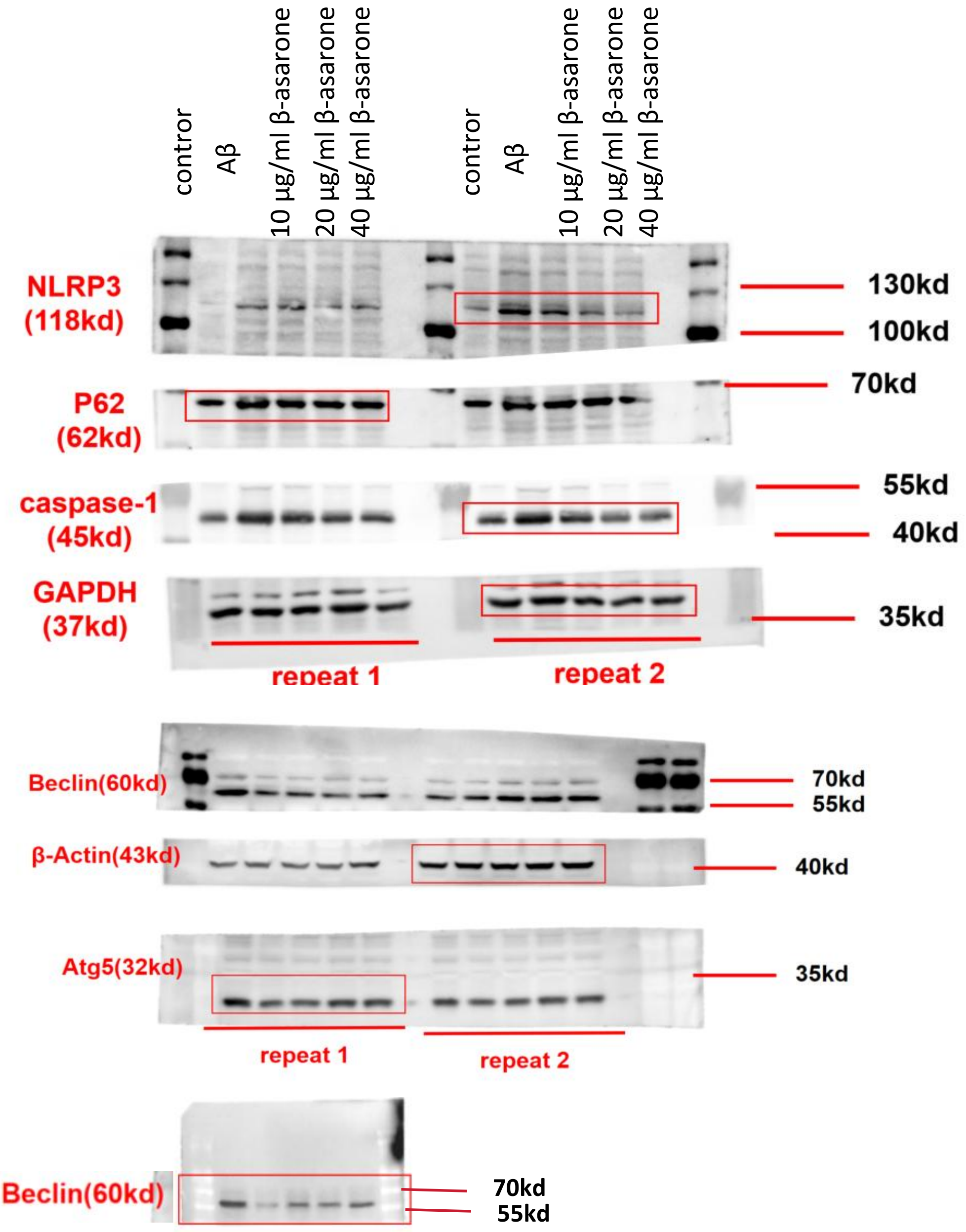

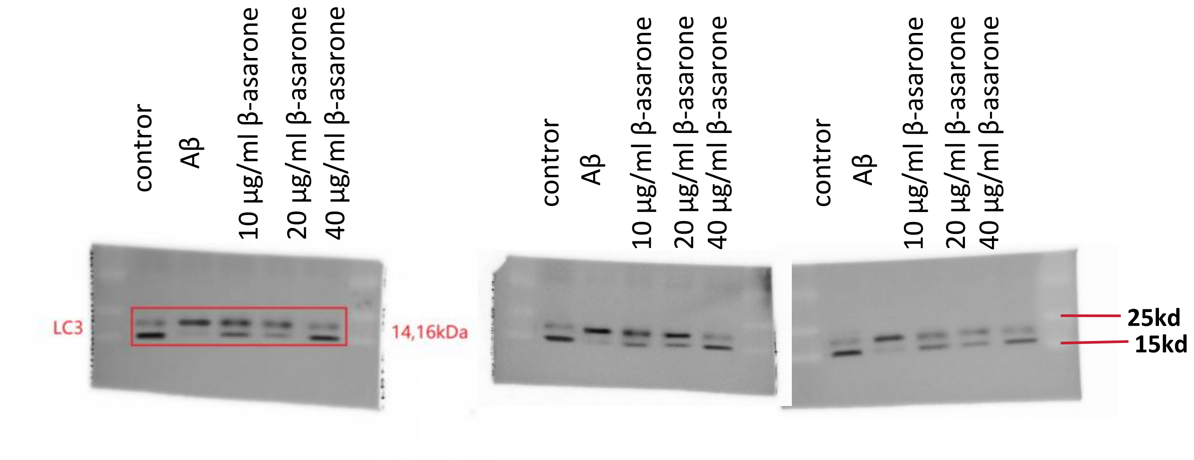

Full unedited gel/blot for Figure 6

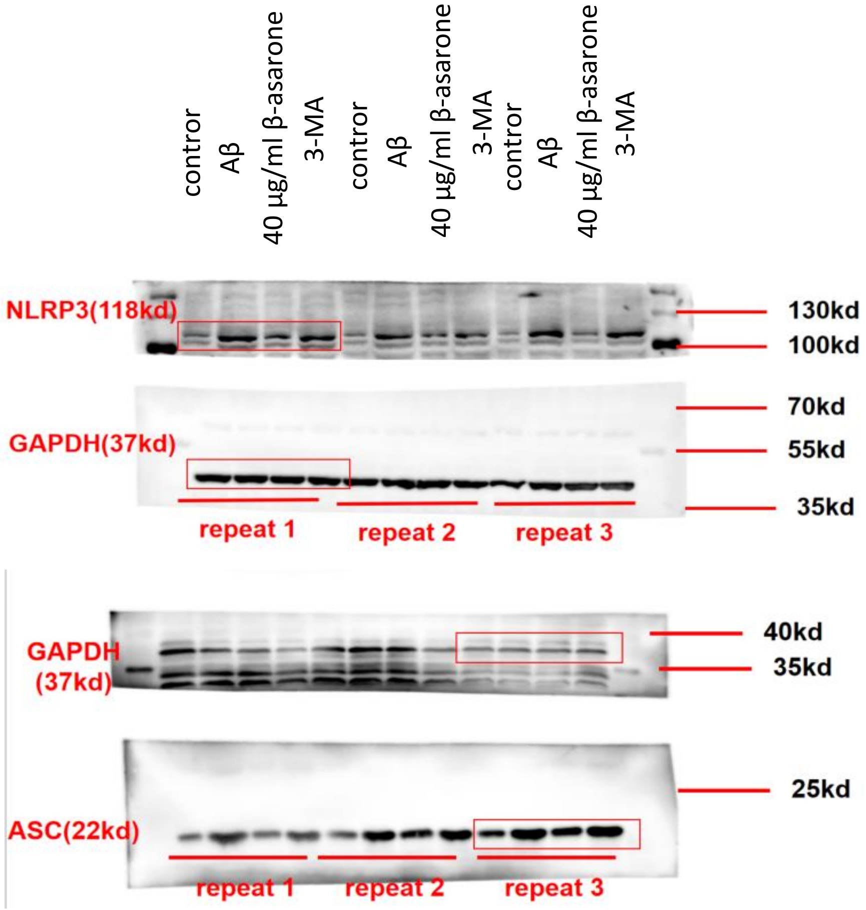

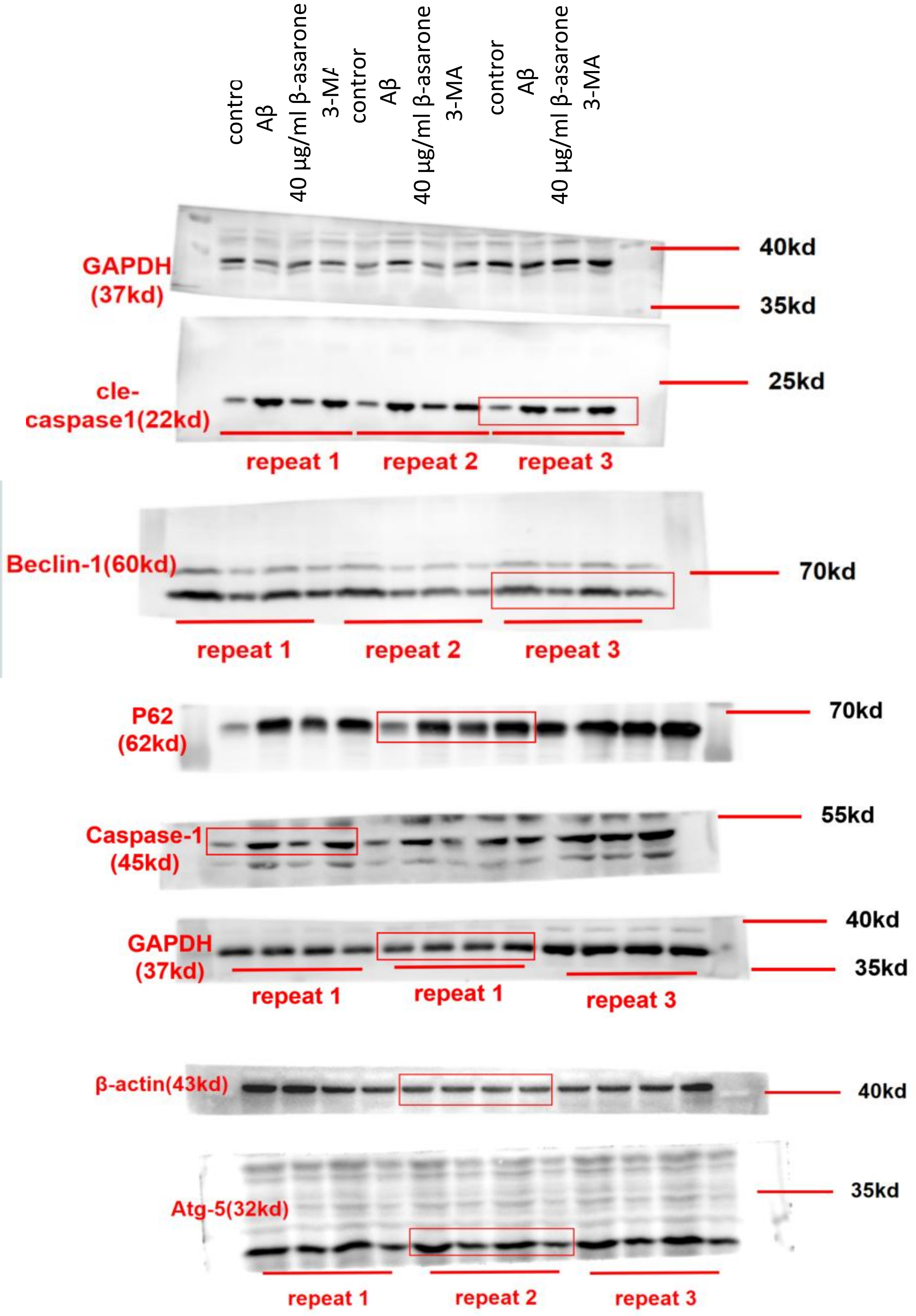

Full unedited gel/blot for Figure 7
