# Supplementary material for: β‐Asarone Attenuates Neuroinflammation of Alzheimer's Disease by Activating Autophagy and Suppressing NLRP3 Inflammasome Assembly
Source: CNS Neurosci Ther. 2026 Feb 4;32(2):e70771. doi: 10.1002/cns.70771 (PMC12869116; doi:10.1002/cns.70771)
Supplement: Supplementary file 1 — Figure S1: Inhibition of NLRP3 ineffectual the activation of β‐asarone on autophagy in microglia induced by Aβ. (A) Representative immunoblots of NLRP3, Beclin‐1 and LC3‐II/I. (B–D) Quantification of relative protein level of NLRP3, Beclin‐1 and LC3‐II/I. Data are expressed as mean ± SD. *p < 0.05, **p < 0.01, relative to contral group; # p < 0.05, ## p < 0.01, relative to Aβ group. n = 3. Aβ = 25 μM, β‐asarone = 40 μg/mL, MCC950 = 10 μM. [file CNS-32-e70771-s001.zip › cns70771-sup-0001-FigureS1.doc]

**Method**

BV-2 cell was obtained from Saibaikang (Shanghai) Biotechnology Co., The cells were cultured in DMEM medium containing 5% FBS and incubated at 37℃ with 5% CO_2._ Cells were exposed to 25 µM Aβ_25-35_ peptide (MedChemExpress, Shanghai, China) for 24 h to establish an Aβ-induced neurotoxicity model. Cells were split into the following categories: control (cells maintained in DMEM only), Aβ group (cells cultured with Aβ_25-35_), β-asarone group (40 μg/ml β-asarone were given for 12 h after Aβ_25-35_ intervention), MCC950 (NLRP3 inhibitor) group (10 μM MCC950 were given for 12 h after Aβ_25-35_ intervention), Combined group (β-asarone and 10 μM MCC950 were treated simultaneously for 12 h after Aβ_25-35_ intervention)

**Result**

Western blot was performed to detect the expressions of NLRP3, Beclin-1 and LC3-Ⅱ/Ⅰ. The experimental results showed that the expressions of NLRP3 in the Aβ group were significantly increased, and went down significantly after treatment with β-asarone, MCC950 and combined application. As for autophagy related indicators Beclin-1 and LC3-Ⅱ/Ⅰ ratio, Beclin-1 expression and LC3-Ⅱ/Ⅰ ratio decreased significantly in the Aβ group, The change trend of these indicators in the Aβ group could be reversed after treatment with β-asarone and combined application. But MCC950 alone suppressed NLRP3 but did not alter LC3-II/I, Beclin-1. β-asarone + MCC950 showed additive NLRP3 inhibition without affecting autophagy markers. Therefore, the results showed that NLRP3 inhibition (via MCC950) did not alter β-asarone-induced autophagy activation.

Figure.S1. Inhibition of NLRP3 ineffectual the activation of β-asarone on autophagy in microglia induced by Aβ. (A) Representative immunoblots of NLRP3, Beclin-1 and LC3-Ⅱ/Ⅰ. (B-D) Quantification of relative protein level of NLRP3, Beclin-1 and LC3-Ⅱ/Ⅰ. Data are expressed as mean±SD. **p* < 0.05, ***p* < 0.01, relative to contral group; ^#^*p* < 0.05, ^##^*p* < 0.01, relative to Aβ group. n=3. Aβ=25 μM, β-asarone=40 μg/ml, MCC950 =10 μM.
